# Cryogenian Origins of Multicellularity in Archaeplastida

**DOI:** 10.1093/gbe/evae026

**Published:** 2024-02-09

**Authors:** Alexander M C Bowles, Christopher J Williamson, Tom A Williams, Philip C J Donoghue

**Affiliations:** School of Geographical Sciences, University of Bristol, Bristol BS8 1SS, UK; Bristol Palaeobiology Group, School of Biological Sciences and School of Earth Sciences, Life Sciences Building, University of Bristol, Bristol BS8 1TQ, UK; School of Geographical Sciences, University of Bristol, Bristol BS8 1SS, UK; Bristol Palaeobiology Group, School of Biological Sciences and School of Earth Sciences, Life Sciences Building, University of Bristol, Bristol BS8 1TQ, UK; Bristol Palaeobiology Group, School of Biological Sciences and School of Earth Sciences, Life Sciences Building, University of Bristol, Bristol BS8 1TQ, UK

**Keywords:** plant evolution, multicellularity, Cryogenian, Archaeplastida, Streptophyta, phylogenetics

## Abstract

Earth was impacted by global glaciations during the Cryogenian (720 to 635 million years ago; Ma), events invoked to explain both the origins of multicellularity in Archaeplastida and radiation of the first land plants. However, the temporal relationship between these environmental and biological events is poorly established, due to a paucity of molecular and fossil data, precluding resolution of the phylogeny and timescale of archaeplastid evolution. We infer a time-calibrated phylogeny of early archaeplastid evolution based on a revised molecular dataset and reappraisal of the fossil record. Phylogenetic topology testing resolves deep archaeplastid relationships, identifying two clades of Viridiplantae and placing Bryopsidales as sister to the Chlorophyceae. Our molecular clock analysis infers an origin of Archaeplastida in the late-Paleoproterozoic to early-Mesoproterozoic (1712 to 1387 Ma). Ancestral state reconstruction of cytomorphological traits on this time-calibrated tree reveals many of the independent origins of multicellularity span the Cryogenian, consistent with the Cryogenian multicellularity hypothesis. Multicellular rhodophytes emerged 902 to 655 Ma while crown-Anydrophyta (Zygnematophyceae and Embryophyta) originated 796 to 671 Ma, broadly compatible with the Cryogenian plant terrestrialization hypothesis. Our analyses resolve the timetree of Archaeplastida with age estimates for ancestral multicellular archaeplastids coinciding with the Cryogenian, compatible with hypotheses that propose a role of Snowball Earth in plant evolution.

**Significance**

Glaciation events of the distant past (720 to 635 million years ago) have been proposed as a driving force for the origin of multicellularity in plants (Archaeplastida). Here, evolutionary analyses produce divergence time estimates for multicellular plants (e.g. red algae and streptophyte algae) spanning this period (the Cryogenian). Our work is compatible with the hypothesis that ancient glaciations facilitated the origin of multicellularity in plants.

## Introduction

The early evolution of the superkingdom Archaeplastida, from the first photosynthetic eukaryotes to the earliest land plants, transformed the biosphere (Lenton et al. 2016; Bengtson et al. 2017; One Thousand Plant Transcriptomes Initiative 2019; Burki et al. 2020; Delaux and Schornack 2021; Schön et al. 2021; Bowles et al. 2022; Irisarri et al. 2022), paving the way for flora that would dominate terrestrial environments. Archaeplastid evolution encompasses a vast period in Earth history, with divergence time estimates spanning the middle Paleoproterozoic to late Neoproterozoic (Sánchez-Baracaldo et al. 2017; Betts et al. 2018; Lutzoni et al. 2018; Morris et al. 2018; Strassert et al. 2021). During this formative period, early archaeplastids influenced the composition of the atmosphere as the dominant primary producers (Brocks et al. 2017; Braakman 2019), provided new ecological niches promoting the diversity of lineages spanning the tree of life (Lutzoni et al. 2018; Del Cortona et al. 2020) and led to the development of more complex food webs (Brocks et al. 2017). Descendants of the archaeplastid crown-ancestor include the rhodophytes, glaucophytes, chlorophytes, streptophyte algae, and land plants (Guiry et al. 2014; Christenhusz and Byng 2016; Corlett 2016; Lughadha et al. 2016), all of which have responded to environmental challenges with novel adaptive mechanisms.

In Archaeplastida, macroscopic organization ranges from filamentous species to siphonous chlorophytes to 3D multicellular organisms, with multicellularity evolving independently on several occasions (Umen 2014). These independent transitions to multicellularity likely occurred during the Neoproterozoic (Knoll 2011; Del Cortona et al. 2020). The Cryogenian (720 to 635 million years ago; Ma) is characterized by two major glaciation events, the Sturtian (717 to 643 Ma) and the Marinoan (656 to 626 Ma) (Hoffman et al. 1998; Stern et al. 2006). These global glaciations, termed Snowball Earth events, encased the Earth in ice (Hoffman et al. 2017), and they have been hypothesized as an evolutionary driver of multicellularity in Archaeplastida, Metazoa, and fungi (Brocks et al. 2017; Del Cortona et al. 2020; Simpson 2021). In particular, Simpson (Simpson 2021) has hypothesized that the 70 million year-long Sturtian glaciation would have led to the near-complete loss of temperate and tropical water habitats, providing a geologically unique, unexplored marine ecological space for the evolution of multicellularity (the Cryogenian multicellularity hypothesis). Seawaters of the Cryogenian would have increased in viscosity, limiting the ability of unicellular life to be motile, feed, and acquire nutrients. Multicellularity may have provided a mechanism to increase motility and size, enabling optimization of nutrient acquisition and metabolic rate (Solari et al. 2006; DeLong et al. 2010).

Cryogenian glaciations have also been proposed as a driver of the colonization of land by early archaeplastids in the Cryogenian Plant Terrestrialization hypothesis (CPT; Becker 2013; Williamson et al. 2019; Žárský et al. 2022). The CPT hypothesis argues that the terrestrial component of snowball Earth environments was a crucial driver for land plant terrestrialization, serving as an intermediary between aquatic and terrestrial habitats. This is because ice environments are dry, exposed, and extremely cold, in contrast to the high viscosity cold sub-glacial seawater habitats. Glacial environments would have exposed ancestral streptophytes to the same environmental challenges that their descendents would have faced in adapting to life on land outside, viz. extreme temperature, irradiance, and desiccation (de Vries and Archibald 2018). Thus, the CPT hypothesis argues that ancestral land plants would have been preadapted to life on land, exapting adaptations evolved on the icy surfaces of Snowball Earth.

Both the Cryogenian multicellularity and CPT hypotheses make specific predictions about the time intervals during which the evolution of multicellularity and terrestrialization, respectively, occurred within Archaeplastida. The Cryogenian multicellularity hypothesis requires that archaeplastid multicellularity evolved coincident with one or more of the Snowball Earth events and the CPT hypothesis requires that land plants evolved in their aftermath. Here, we examine whether these hypotheses are consistent with the timescale of archaeplastid evolution using phylogenetics, divergence time estimation, and ancestral trait reconstruction. While previous studies have aimed to understand the relationships and timescale of green plant evolution, these have often focused specifically on chlorophyte (Del Cortona et al. 2020; Nie et al. 2020; Hou et al. 2022; Yang et al. 2023) and streptophyte algae (Wickett et al. 2014; Morris et al. 2018; Puttick et al. 2018; Hess et al. 2022). Additional work has investigated the phylogeny and divergence times of nongreen plant lineages but at higher taxonomic resolution (e.g. eukaryotes) and with shallower species sampling (Sánchez-Baracaldo et al. 2017; Betts et al. 2018; Lutzoni et al. 2018; Strassert et al. 2021). This work incorporates both a breadth and depth of taxonomic sampling, spanning rhodophytes, glaucophytes, chlorophytes, and streptophyte algae.

We first inferred a rooted phylogeny of Archaeplastida, using both concatenation- and coalescent-based approaches, combined with tree topology testing. We then estimated the timescale of archaeplastid evolution, incorporating an updated appraisal of the fossil record and a broad taxonomic sampling of genes. Finally, we reconstructed the evolution of multicellularity across Archaeplastida, demonstrating that their timing coincides with the Cryogenian. These results are compatible with the hypotheses that environmental conditions during Cryogenian Snowball Earth glaciations underpinned the evolution of archaeplastid multicellularity and the exaptive evolution of land plant innovations envisaged by the CPT hypothesis.

## Results

### Phylogenomics Resolves the Relationships of Archaeplastida

**Fig. 1.**
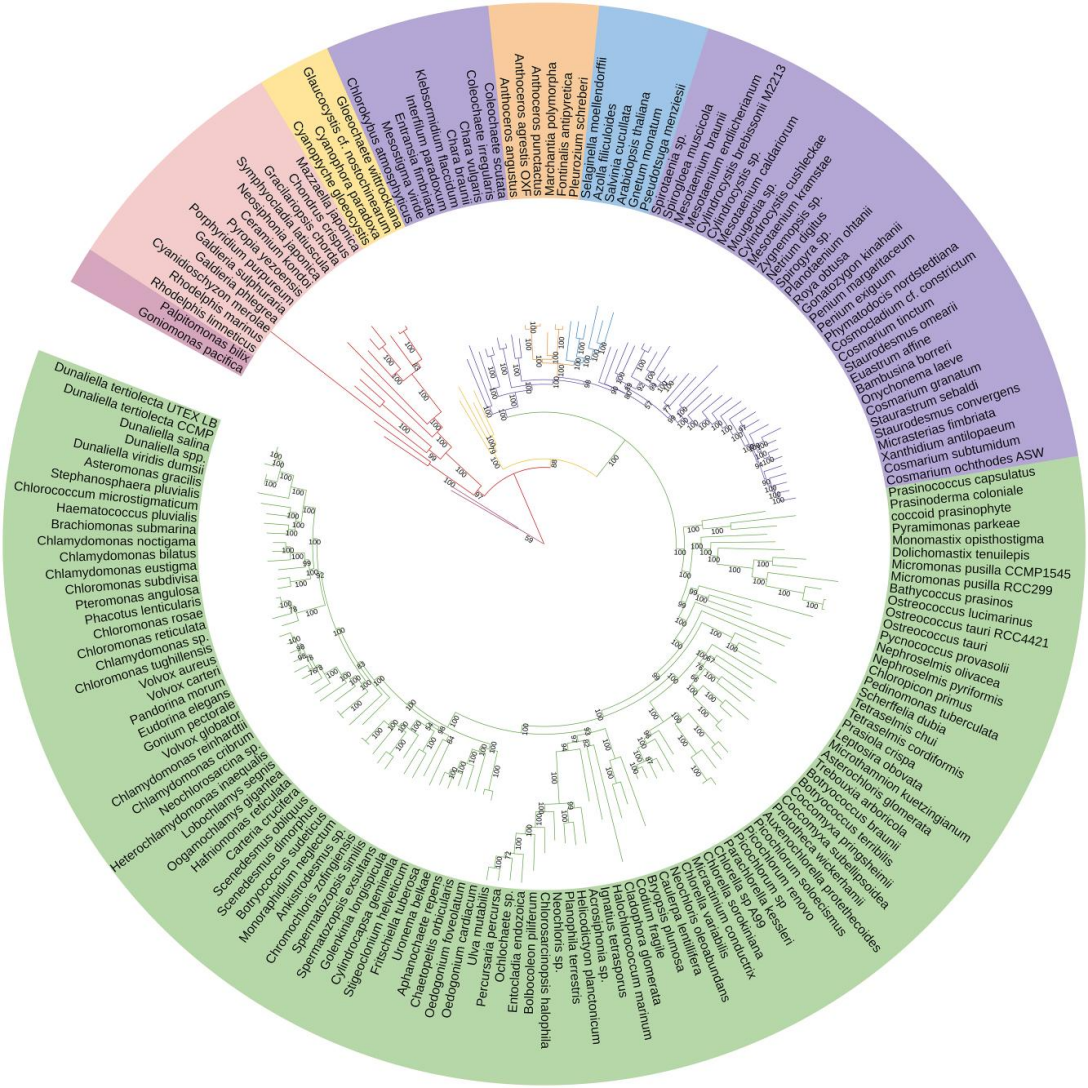
Phylogeny of Archaeplastida based on concatenation-based analysis of 96 genes representing 180 species. Branches and taxa are grouped into Outgroup (red group including *Palpitomonas bilix*), Rhodophytes (pink group including *Chondrus crispus*), Glaucophytes (yellow group including *Cyanophora paradoxa*), Chlorophytes (green group including *Volvox carteri*), Streptophyte algae (purple group including *Chara braunii* and *Spirogloea muscicola*), Bryophytes (blue group including *Marchantia polymorpha*) and Tracheophytes (orange group including *Arabidopsis thaliana*).

Recent genome and transcriptome sequencing of under-represented lineages can be utilized to address poorly resolved nodes in the archaeplastid phylogeny. We built a comprehensive dataset of 241 species, with improved representation for previously under-sampled lineages, most notably streptophytes, early diverging chlorophytes, and rhodophytes (supplementary data S1, Supplementary Material online, supplementary information S1, Supplementary Material online). Maximum likelihood analysis, based on a concatenation of 96 genes using the best-fitting model for each gene partition, resolves the fundamental split within Archaeplastida between rhodophytes and glaucophytes plus Viridiplantae, recovers two clades of green plants (chlorophytes and streptophytes), and supports Bryopsidales as sister group to the chlorophyceae (Fig. 1). Our phylogenetic analyses also support the monophyly of Archaeplastida, the sister group relationship between Zygnematophyceae and land plants (Anydrophyta) and the monophyly of bryophytes and tracheophytes (Embryophyta; Fig. 1), in agreement with recent work (Wickett et al. 2014; Puttick et al. 2018; Gawryluk et al. 2019; One Thousand Plant Transcriptomes Initiative 2019; Price et al. 2019; Harris et al. 2020). These relationships were also supported in our coalescent-based analyses (supplementary information S2, Supplementary Material online).

**Fig. 2.**
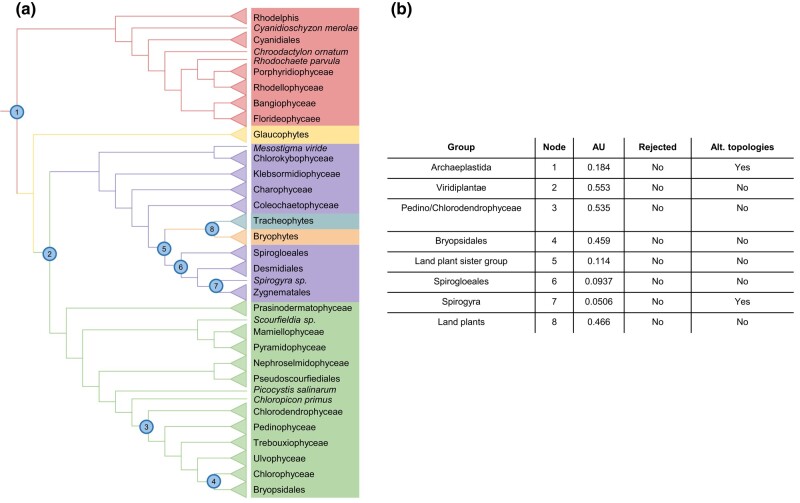
Support for alternative topologies of the Archaeplastida phylogeny. a) Phylogeny of Archaeplastida with key nodes numbered. b) Support for focal and alternative topologies for all major nodes in the Archaeplastida phylogeny.

Phylogenetic conflict associated with contested relationships was addressed using approximately unbiased (AU) tests (Fig. 2), allowing for evaluation of the support for the recovered and alternative phylogenetic relationships. The relationships recovered receive support based on this analysis, while previously proposed alternative topologies for Prasinodermophyceae (Li et al. 2020) and Bryopsidales (Fang et al. 2017) were not supported by the AU test. Several relationships remain enigmatic, with either support for multiple competing topologies or conflicting support across datasets (Fig. 2). Overall, these analyses provide a robust framework to date early archaeplastid evolution.

### The Timescale of Early Archaeplastid Evolution

**Fig. 3.**
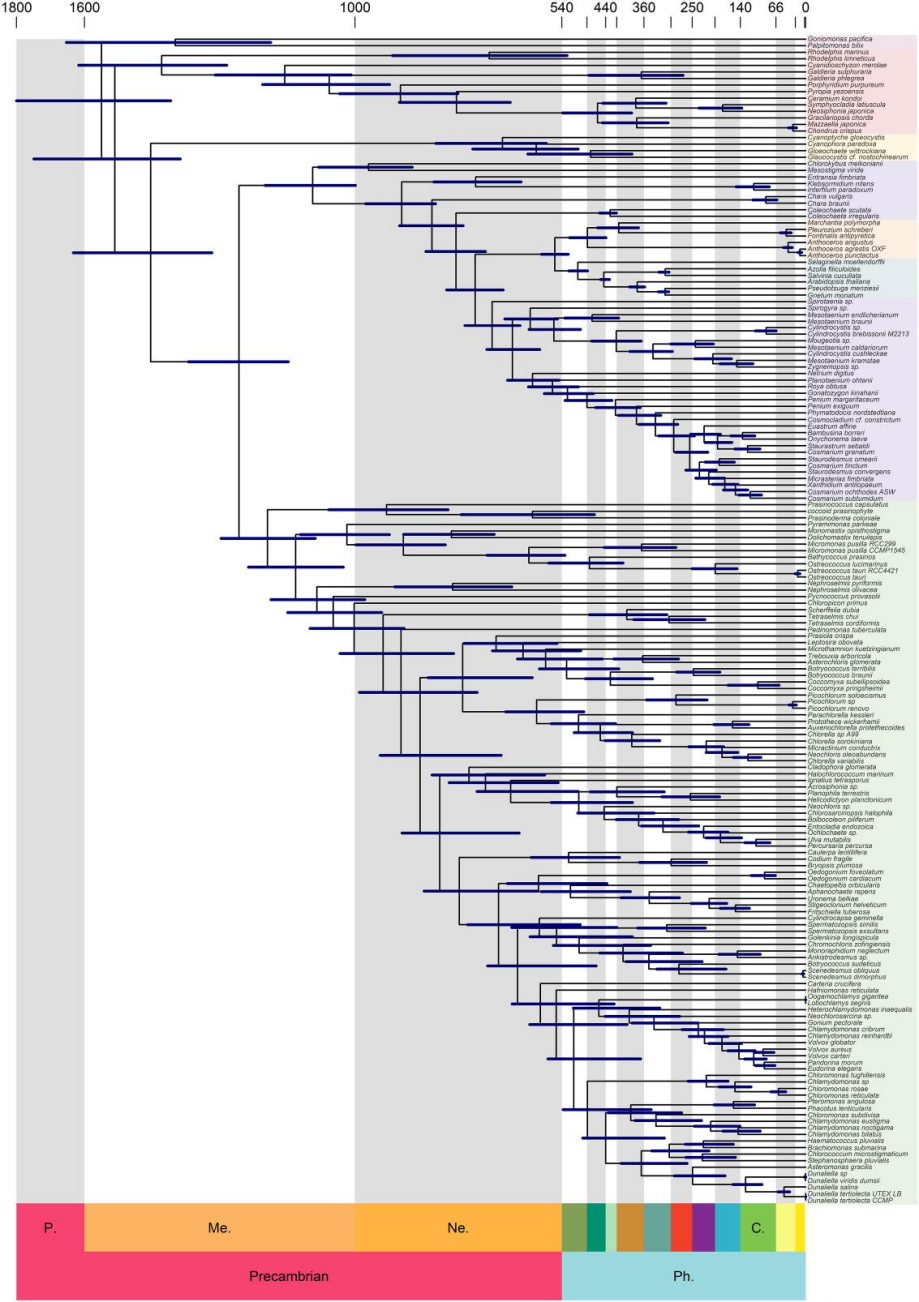
Time-calibrated phylogeny of Archaeplastida with 95% highest posterior density age uncertainties. Taxa are grouped into Outgroup (red group including *Palpitomonas bilix*), Rhodophytes (pink group including *Chondrus crispus*), Glaucophytes (yellow group including *Cyanophora paradoxa*), Chlorophytes (green group including *Volvox carteri*), Streptophyte algae (purple group including *Chara braunii* and *Spirogloea muscicola*), Bryophytes (blue group including *Marchantia polymorpha*) and Tracheophytes (orange group including *Arabidopsis thaliana*).

We estimated divergence times along the resolved phylogeny of Archaeplastida (Fig. 3), aided by a dataset of 15 fossil calibrations (supplementary information S3, Supplementary Material online). The red alga, *Bangiomorpha pubescens* and the green alga, *Proterocladus antiquus* are considered as the oldest convincing records of Archaeplastida (See Materials and Methods for further discussion on archaeplastid fossils). Within the multicellular land plants, robustly supported fossil calibrations were identified for the major nodes in the embryophyte phylogeny.

Our results support an origin of Archaeplastida during the Mesoproterozoic (1,712 to 1,387 Ma), while crown-rhodophytes, crown-glaucophytes, and crown-Viridiplantae emerged 1,613 to 1,284 Ma, 1,624 to 1,317 Ma, and 1,369 to 1,147 Ma, respectively (Fig. 3). Among Viridiplantae, crown-chlorophytes diverged by 1,297 to 1,088 Ma while crown-streptophytes diverged by 1,199 to 998 Ma (late Mesoproterozoic—earliest Neoproterozoic). Within the streptophytes, Phragmoplastophyta emerged 901 to 760 Ma, crown-Anydrophyta (the clade of Zygnematophyceae and land plants) diverged 796 to 671 Ma, while crown-Embryophyta emerged 586 to 526 Ma in a late Ediacaran to Cambrian interval.

To explore the impact of different models of substitution and rate heritability on divergence time estimation, we undertook analyses using the CAT-GTR model in Phylobayes and the correlated rates model in MCMCTree, respectively (supplementary informations S6 and S7, Supplementary Material online). These different approaches produced divergence time estimates that were largely congruent with our main analysis. For example, the correlated rates analysis recovered a divergence time for crown-Anydrophyta of 742 to 654 Ma whilst the initial analysis recovered an estimate of 796 to 671 Ma. These results suggest that the divergence timescale derived from our main analysis, using the uncorrelated rates model in MCMCTree, is robust of these methodological variables.

### Independent Emergence of Multicellular Archaeplastids

**Fig. 4.**
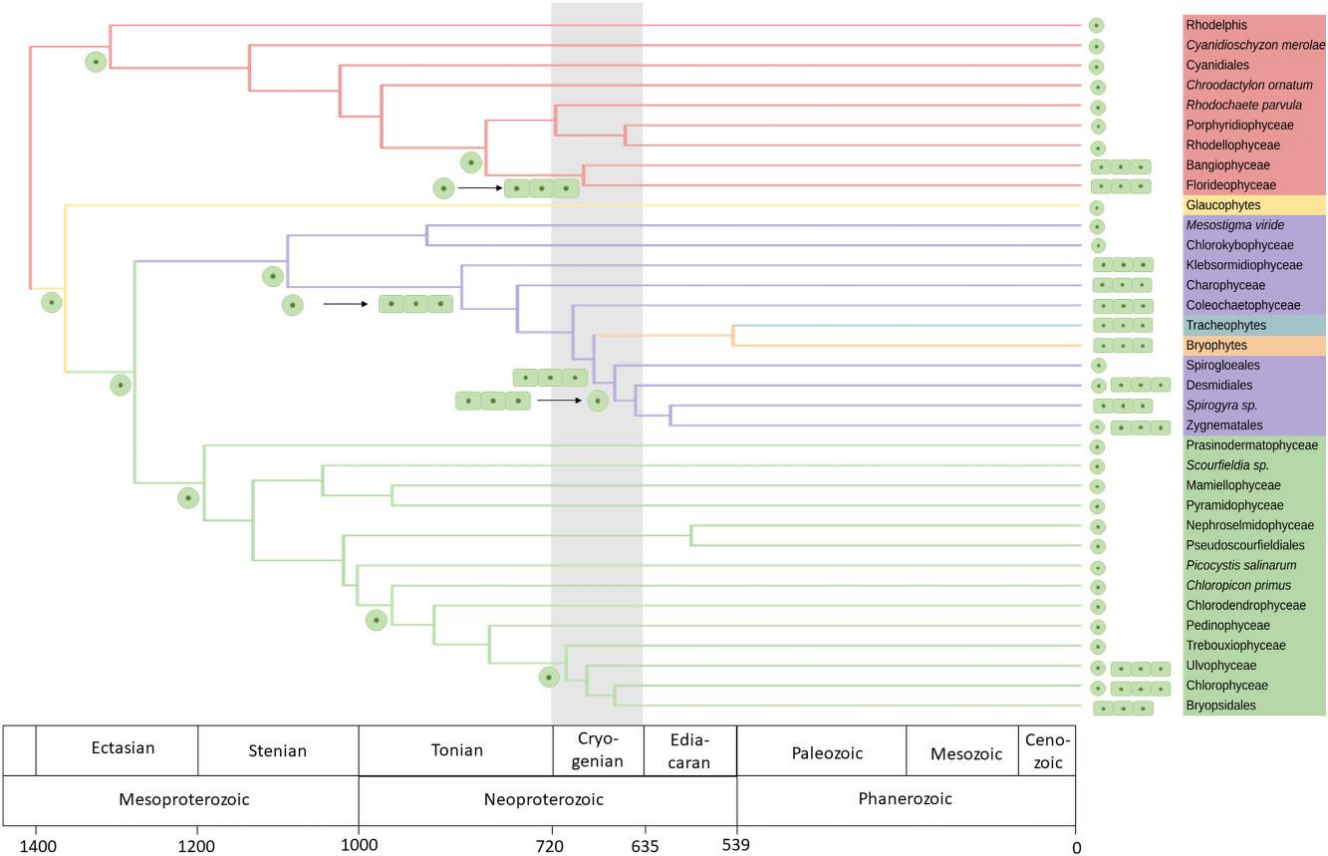
Ancestral state reconstruction of cytomorphological traits across the Archaeplastida. The topology of the tree is based on collapsed branches of the analysis presented in Fig. 3. Branch lengths are based on age estimates from the timescale analysis presented in Fig. 3. Ancestral state plotted onto the tree are summarized from supplementary information S5b, Supplementary Material online.

Stochastic mapping of ancestral states of multicellularity in early archaeplastids revealed that multicellularity in streptophytes and rhodophytes was present in the ancestors of Anydrophyta and Eurhodophytina, respectively (Fig. 4, supplementary informations S4 and S5, Supplementary Material online). Taking our timescale analysis into consideration, the radiation of these two multicellular lineages spans the Cryogenian (720 to 635 Ma). The diversification of chlorophytes was accompanied by multiple origins of multicellularity (e.g. Bryopsidales, Chaetophorales, and Oedogoniales). The diversification of multicellular Chaetaophorales (661 to 441 Ma) possibly occurred during the Cryogenian while the radiation of multicellular Bryopsidales (609 to 413 Ma) and Ulvales (364 to 237 Ma) occurred more recently.

## Discussion

### Comparison to Previous Studies

We present a time-calibrated phylogeny of archaeplastid evolution using a large-scale multigene dataset and an up to date suite of fossil calibrations. Modeling the evolution of cytomorphology within this framework reveals multiple, independent transitions from unicellular to multicellular growth in archaeplastids during the Neoproterozoic (Fig. 4). Recent studies have resulted in contradictory phylogenies. The phylogeny from the One Thousand Plant Transcriptome Project (1KP project) recovered Prasinococcales as sister to streptophytes (One Thousand Plant Transcriptomes Initiative 2019) while analysis of the *Prasinoderma coloniale* genome recovered Prasinococcales as the sister group to all other green plants (Li et al. 2020). The 1KP project also recovered support for two positions for Bryopsidales while recent phylogenomic analyses found support for contrasting positions for Bryopsidales between analytical approaches (One Thousand Plant Transcriptomes Initiative 2019; Del Cortona et al. 2020). Maximum likelihood analysis of the *Spirogloea muscicola* genome inferred Spirogloeales as an early branching lineage within Zygnematophyceae, although Bayesian inference pointed to a potential sister group relationship between Spirogloeales and land plants (Cheng et al. 2019). Our phylogenetic analyses, both concatenation- and coalescence-based, mark a significant consolidation of recent thinking on archaeplastid evolution, addressing some long-standing questions including the grouping of green plants and the nonmonophyly of Ulvophyceae. Although our study focuses on the early evolution of Archaeplastida, the relationships within land plants (e.g. monophyly of bryophytes) agree with the recent phylogenetic analysis (Puttick et al. 2018; One Thousand Plant Transcriptomes Initiative 2019; Harris et al. 2020, 2022), providing additional support for these formerly uncertain regions of the archaeplastid tree.

Given the deep evolutionary history of early archaeplastid evolution, previous analyses have wide-ranging divergence time estimates. The origin of Archaeplastida has been estimated to the middle Palaeoproterozoic to early Mesoproterozoic interval (2137 to 1118 Ma; 5,7 to 9,11). Our analysis refines these estimates, recovering a divergence time of 1712 to 1387 Ma, spanning the late-Paleoproterozoic to early-Mesoproterozoic. Viridiplantae has been estimated to emerge between the middle Mesoproterozoic to middle Neoproterozoic (1,400 to 669.9 Ma; 5,7,10,11) with our analysis recovering a divergence time of 1.369 to 1,147 Ma, during the mid-late Mesoproterozoic. Previous analyses have dated the origin of the first streptophytes during the middle Mesoproterozoic to late Neoproterozoic era (1,340 to 629.1 Ma; 5,10,11). Our analysis recovers a divergence time of 1,199 to 998 Ma (late Mesoproterozoic—earliest Neoproterozoic). These differences between our study and previously published work are likely due to taxon sampling as well as the interpretation of the fossil record, particularly for studies with taxa from the rhodophytes, glaucophytes, and green plants (Sánchez-Baracaldo et al. 2017; Betts et al. 2018; Lutzoni et al. 2018; Strassert et al. 2021).

Stochastic mapping, based on the time-calibrated phylogeny, is consistent with the Cryogenian multicellularity hypothesis, in which global glaciations promoted the origin of multicellularity across the eukaryotes (Fig. 4). Our timescale is compatible with this hypothesis, at least for red and streptophyte algae, though show contrasting patterns for the chlorophytes. For the latter, our data indicate that the Cryogenian multicellularity hypothesis is plausible for the ancestor of Chaetophorales and Oedogoniales. However, we show here that multicellularity within Bryopsidales and the Ulvophyceae originated in the Phanerozoic.

Stochastic mapping estimates ancestral states to a given node (crown group); however, the evolution of multicellularity may predate this ancestor (stem group). Therefore, while our analysis has localized the evolution of multicellularity to particular stem branches, the precise point of these state transitions is difficult to pinpoint. As such, while our analysis can provide a confident most recent divergence time of multicellular groups, there is a degree of uncertainty about the upper end of these estimates. This uncertainty may affect the potential temporal relationship between the origins of multicellularity and snowball Earth. The coding of characters also has important implications for ancestral character states. The coding of characters did not dramatically change the ancestral states in streptophyte and red algae. However, this did impact the ancestral state reconstruction in the chlorophyte algae, with the approaches coding more divisions of multicellularity (siphonous, sarcinoid, etc.) led to separate, distinct origins of multicellular groups (supplementary information S5a and b, Supplementary Material online). When these divisions of multicellularity (siphonous, sarcinoid, etc.) were placed within a single multicellular group, then multicellularity emerged once in the ancestor of Ulvophyceae and Chlorophyceae (supplementary information S5c, Supplementary Material online). Analysis of the posterior probability density of this binary definition of multicellularity highlights the increasing likelihood of a multicellular ancestor along the spine of the streptophyte phylogeny (supplementary information S5d, Supplementary Material online).

### Multicellularity and the Cryogenian

With multiple origins across the tree of life as well as multiple definitions, the transition to multicellularity is not a simple story (Rose and Hammerschmidt 2021). Here, we use a broad definition of multicellularity to encompass any nonunicellular species. While these nonunicellular species are found across our phylogeny, complex multicellular organisms have traditionally been classified in only two clades of Archaeplastida, florideophyte red algae, and land plants (Knoll 2011). Our results suggest a stepwise evolution leading to more complex multicellularity in both these lineages. Our timescale is compatible with the interpretation of diverse taxa in the Ediacaran Weng’an biota (635 to 541 Ma), as stem-Corallinales (Xiao et al. 1998).

The paucity of the streptophyte algal fossil record prevents morphological comparisons, but phylogenetic analysis provides mechanistic insights. The evolution of multicellularity required changes to gene networks regulating cell to cell adhesion, intercellular communication and cellular differentiation (Niklas 2014; Niklas and Newman 2020). The xyloglucan endotransglucosylase/hydrolases gene family, involved in plant cell wall expansion, were horizontally transferred from Alphaproteobacteria to the ancestor of Phragmoplastophyta and Klebsormidiophyceae and subsequently diversified in younger lineages (Shinohara and Nishitani 2021). Importantly, this gene was transferred during the Cryogenian. Several novel genes involved in cellulose synthesis, and therefore cell wall construction, emerge in the ancestor of Phragmoplastophyta and Klebsormidiophyceae (Lampugnani et al. 2019). Indeed, genome sequencing of zygnematophyte genomes indicated that most of the enzymes for cell wall innovations were present in this ancestor (Feng et al. 2023). Genome analysis has demonstrated how proliferations of genes involved in phytohormone signaling and cellular development lead to the emergence of the phragmoplast, a cytoplasmic structure involved in cell division, in the streptophyte lineage Phragmoplastophyta (Umen 2014; Nishiyama et al. 2018). Further genetic diversification and origin of new genes in Anydrophyta led to the emergence of all major enzymes for the synthesis of cell wall components (Feng et al. 2023). The findings from the literature above and our analysis of the divergence time of Archaeplastida correlates the emergence of these genes to the Cryogenian, providing an initial mechanistic basis of multicellular adaptations.

Recent biomarker analysis has suggested that archaeplastid algal lineages became dominant over cyanobacteria after the Sturtian glaciation (Brocks et al. 2017; Hoshino et al. 2017; Brocks 2018). Some recent work has conflated the emergence of crown group eukaryotes with their early diversification (Porter et al. 2018; Budd and Mann 2020; Porter 2020), therefore proposing much later divergence time estimates (e.g. the Mesoproterozoic–Neoproterozoic boundary). However, our analysis identifies a distinction between the origin of photosynthetic eukaryotes in the late-Paleoproterozoic to early-Mesoproterozoic and their rise to ecological dominance (Figs. 3 and 4). Our analyses do not directly address when algae achieved ecological dominance though we note that there are alternative explanations of the biomarker record and that ecological modeling suggests they achieved significance early in the Proterozoic (Eckford-Soper et al. 2022).

Alongside environmental factors of the Snowball Earth conditions during the Cryogenian, several other drivers of multicellularity have also been proposed, including oxygen concentration, predation and nutrient concentration (Mills and Canfield 2014; Brocks et al. 2017; Herron et al. 2019). The ability to extract increased oxygen and nutrients, via multicellularity, may have provided ancestral organisms with competitive advantages in ancient environments (Knoll 2011; Brocks et al. 2017) while selection imposed by predators may have contributed to the emergence of multicellularity (Herron et al. 2019). These may have been additional important factors contributing to the colonization of snowball Earth by ancestral multicellular algae.

### Plant Terrestrialization and the Cryogenian

The estimate of 796 to 671 Ma for the divergence of Anydrophyta is entirely compatible with the Cryogenian plant terrestrialization hypothesis (Fig. 3). While temporal co-occurrence is distinct from causality, an origin for Anydrophyta during the Cryogenian is intriguing in the light of ancient cryospheric environments which are hypothesized as a major factor in the selection of key biological adaptations to terrestrial environments, leading ultimately to the origin of crown-embryophytes in the earliest Phanerozoic. This indicates that the time between the divergence of Embryophyta from Zygnematophyceae and the divergence of bryophytes from tracheophytes (i.e. the Embryophyte stem-lineage) was between 270 and 85 Myr in duration. In either instance, this is a long time span in which to assemble the body plan of crown-embryophytes, including the acquisition of embryonic development, 3D growth, cuticle, stomata, meiospores and, likely, vascular tissues and axial branching (Donoghue et al. 2021). At the least, our ancestral state estimation indicates that the streptophyte lineage was already multicellular before the origin of Phragmoplastophyta, providing formal trait-based inference that Zygnematophyceae are secondarily simplified, corroborating recent inferences that these algae have lost many morphological traits (Buschmann and Zachgo 2016; Moody 2020; Hess et al. 2022) and genes since their common ancestor with land plants (Cheng et al. 2019; Bowles et al. 2020; Jiao et al. 2020; Gong and Han 2021; Harris et al. 2022). It remains unclear in which environment and in what order these novelties arose. Extant Zygnematophyceaen algae (e.g. *Ancylonema nordenskiöldii* and *A. alaskana*) inhabit modern glaciers and are found in the sister lineage to land plants (Williamson et al. 2019; Procházková et al. 2021). Genome analysis of extant glacier algae will provide insight into these ancient physiological adaptations.

The origin of multicellular archaeplastids transformed the atmosphere, with larger organisms undertaking higher rates of photosynthesis, consuming more carbon dioxide and producing more oxygen (Simpson 2021). This evolutionary innovation led to ancestral archaeplastids becoming the dominant contributor to net primary production in ancient environments, surpassing cyanobacteria (Brocks et al. 2017). The consequent increase in atmospheric oxygen likely led to the development of new ecological niches and increased efficiency in the transfer of nutrients and energy between complex trophic structures. Elevated energy availability drove the rise of increasingly complex organisms and subsequently ecosystems. Ultimately, these drastic evolutionary changes would provide the backdrop to the origin of the first land plants, one of the most profound geobiological events in Earth history.

## Materials and Methods

### Dataset Selection

We used a latest sampling of transcriptome and genome data to infer the evolutionary history of Archaeplastida. Specifically, these included nonembryophyte transcriptomes from the one thousand plant transcriptomes project and genomes across the archaeplastid tree of life (supplementary information S1, Supplementary Material online, supplementary data S1, Supplementary Material online). Taxonomic sampling and genome completeness are key factors influencing the reconstruction of phylogeny. Considering this, distinct datasets were curated focusing on different branches of early archaeplastid evolution and the quality of the input data. These different datasets were used to assess the robustness of recovered topologies. On the whole, topologies across datasets were congruent. In total, eight datasets were assembled, focusing on Streptophyta, Viridiplantae, and Archaeplastida with and without outgroup taxa. For each of these taxonomic groups, a data rich and taxon rich dataset were assembled. For the data rich datasets, BUSCO analysis, with the Eukaryota dataset, was used to assess the quality of transcriptome and genome data (Waterhouse et al. 2018) (supplementary data S1, Supplementary Material online). A benchmark of 30% missing BUSCO genes was used to filter high quality data. For the taxon rich dataset, all taxa were included. Therefore, the datasets consist of Streptophyta data rich (57 species), Streptophyta taxon rich (64 species), Viridiplantae data rich (166 species), Viridiplantae taxon rich (203 species), Archaeplastida without outgroup data rich (178 species), Archaeplastida without outgroup taxon rich (239 species), Archaeplastida data rich (180 species), and Archaeplastida taxon rich (241 species) (supplementary data S1, Supplementary Material online).

### Orthology Inference

OrthoFinder (Version 2.3.7) was used to cluster protein coding genes into orthogroups (Emms and Kelly 2019), using default settings (orthofinder -f data_folder). For all datasets, no universally present single copy orthologs were identified. Single copy genes are important for accurate phylogenetic inference. Therefore, near-single copy orthologs were identified using a previously described python script (Harris et al. 2020) which removes paralogous genes from orthogroups. The script enables the user to specify a minimum taxonomic occupancy of each orthogroup, set at 80%. Therefore, the datasets consist of Streptophyta data rich (222 genes), Streptophyta taxon rich (122 genes), Viridiplantae data rich (204 genes), Viridiplantae taxon rich (134 genes), Archaeplastida without outgroup data rich (133 genes), Archaeplastida without outgroup taxon rich (69 genes), Archaeplastida data rich (96 genes), and Archaeplastida taxon rich (48 genes).

### Species Tree Inference

#### Supermatrix Analysis

Single copy orthologs were aligned using MAFFT (Katoh et al. 2002) using –auto parameter and trimmed with Trimal (Capella-Gutiérrez et al. 2009) using the –automated1 parameter. Multiple sequence alignments were concatenated using Phyutility to create a supermatrix (Smith and Dunn 2008). A bootstrapped maximum likelihood phylogeny was inferred using IQ-Tree (Nguyen et al. 2015) using the Bayesian Information Criterion (BIC) to select best-fitting substitution model including empirical profile mixture models (C10–C60), which allow for compositional heterogeneity across sites and are much less prone to mutational saturation than simple single-matrix models (Williams et al. 2021). 1,000 ultrafast bootstrap replicates were used.

#### Supertree Analysis

Individual maximum likelihood gene trees were inferred for each single copy gene using IQ-Tree (Nguyen et al. 2015), using the BIC to select the best-fitting substitution model including empirical profile mixture models (C10–C60), which allow for compositional heterogeneity across sites. Supertree analysis was conducted using ASTRAL (Zhang et al. 2018) with default settings (supplementary information S2, Supplementary Material online).

### Testing the Proposed Relationships of Early Archaeplastids

Uncertain and contested relationships across the early archaeplastid phylogeny were identified (Fig. 2b), including the position of the Zygnematophyceae, Bryopsidales, Prasinodermatophyta, Glaucophytes, and Rhodophytes (One Thousand Plant Transcriptomes Initiative 2019). Phylogenies were then constrained in IQ-Tree with these possible topologies and the support for these relationships was assessed using the Kishino and Hasegawa (Kishino and Hasegawa 1989; Goldman et al. 2000), Shimodaira and Hasegawa (Shimodaira and Hasegawa 1999), and AU (Shimodaira 2002) tests built within IQ-Tree. Based on these inferences, unlikely species tree hypotheses could then be rejected (Fig. 2).

### Testing for the Impact of Across-branch Compositional Heterogeneity

Due to the deep evolutionary relationships of Archaeplastida, we tested the impact of compositional heterogeneity in our dataset. We used a previously described python script (Williams 2023), to filter out the 50% most compositional heterogeneous amino acid sites in our alignment. Phylogenetic analysis, using the methods from our supermatrix analysis, was repeated for this reduced dataset. This recovered the same topology suggesting our analysis is based a phylogenetically informative alignment (supplementary information S8, Supplementary Material online).

### Divergence Time Estimation

We undertook a critical review of the fossil evidence used to calibrate archaeplastid evolution (see calibration descriptions in the supplementary information S3, Supplementary Material online). Interpreting the fossil record of early archaeplastids is challenging as the remains of unicellular algae are simple, precluding confident assignment to extant clades, while fossilized multicellular algae are often difficult to discriminate from filamentous cyanobacteria (e.g. *Archaeophycus yunnanensis* has been alternately interpreted as a bangiophyte red alga (Xiao et al. 1998), a trebouxiophyte green alga (Shang and Liu 2022) or a cyanobacterium (Pan et al. 2022). With this in mind, 15 calibrations were assigned based on stringent criteria (Parham et al. 2012), with the most influential being the multicellular green algae, *Proterocladus antiquus* (Gibson et al. 2018), used to calibrate the ancestor of Viridiplantae and the multicellular red algae, *Bangiomorpha pubescens* (Tang et al. 2020), used to calibrate the ancestor of Archaeplastida (supplementary Information S3, Supplementary Material online). While we are confident in the assignment of these fossils to Chlorophyta and Rhodophyta (respectively), their assignment to more derived lineages within these clades is based on their general gestalt, not on evidence of a nested hierarchy of characters that would support their assignment within the crown clades of red or green algae (Bowles et al. 2022). Similarly, a suite of fossil taxa from the Ediacaran Weng’an biota have been assigned to Bangiophyta and to derived clades within Florideophyta (Xiao et al. 2004, 1998) and they have been used widely to calibrate these clades (e.g. Yoon et al. 2004; Parfrey et al. 2011; Yang et al. 2016); unfortunately, they lack key phenotypic synapomorphies of crown-Rhodophyta (e.g. Gabrielson et al. 1985). We therefore interpret these taxa as records of the rhodophyte and chlorophyte total groups, reflecting uncertainty over whether they are member of their respective stem- or crown-groups. As we map the transition of multicellularity in rhodophytes to the common ancestor of Bangiophyceae and Florideophyceae (see results), this could be used to argue that these fossils should be used to calibrate a more derived position within red and green algae. However, it is difficult to exclude the loss of phenotypic features as well as extinction of evolutionary intermediates, hence our conservative use of the fossil record. Other possible early records of Archaeplastida (e.g. *Rafatazmia chitrakootia, Ramathallus lobatus, Tawuia*, and many unicellular algae) (Bengtson et al. 2017; Tang et al. 2021; Moczydłowska and Liu 2022) are too uncertain to be used as calibrations, at least at present (Carlisle et al. 2021; Bowles et al. 2022).

Node distributions using minimum and maximum constraints were specified, with full phylogenetic and age justifications listed in supplementary Information S3, Supplementary Material online. To specify the prior distributions on node ages, all calibrated land plant nodes were given a hard minimum age and a soft maximum age. For all algal calibrations, as their dates are harder to restrict, a 2.5% probability of exceeding both the minimum and maximum was used.

Initially, analyses were run without sequence data to obtain effective time priors, to ensure that the calibration densities and time priors were appropriate. A dataset of 164 protein coding genes were identified based on a taxonomic occupancy of 78% amongst the Archaeplastida data rich taxa. The single copy orthogroups were divided into 4 partitions according to their evolutionary rate, based on total tree length in IQTree (Nguyen et al. 2015) and grouped using k-means clustering in R (R Core Team 2023). A log-normal independent relaxed clock model was used. Given the protein coding gene dataset, branch lengths were first estimated using codeml (Yang 2007). The gamma distribution for the mean substitution rate was assigned a diffuse shape parameter of 2 and a scale parameter of 10, based on pairwise distance between *Arabidopsis thaliana* and *Marchantia polymorpha*, assuming a divergence time of 469 Ma (Morris et al. 2018). The tree topology was fixed based on the focal maximum likelihood analysis above and was analyzed using the normal approximation method in MCMCtree (Yang 2007). The rate variation was assigned a shape parameter of 1 and a scale parameter of 10. The birth and death parameters were set to 1, specifying a uniform kernel. Burn-in was set at 40,000 with 400,000 samples and sampling frequency of 5. Two independent runs were completed. Trees were plotted using MCMCTreeR (Puttick 2019). A full list of posterior divergence time estimates are summarized in supplementary informations S9 and S10, Supplementary Material online.

### Comparison to Other Approaches

The impact of correlated rates on divergence times was assessed within MCMCTree, whilst all other parameters and input data remained the same as above. These were plotted in MCMCTreeR (supplementary Information S6, Supplementary Material online). Analysis was also conducted in Phylobayes (4.1: Lartillot et al. 2009), under log-normal autocorrelated relaxed clock models, using site-heterogenous CAT + GTR + G models. The same input data, tree topology and fossil calibration strategies were used as the main MCMCTree analysis. Two independent MCMC chains were run, the first 10% discarded as burnin. These were plotted in MCMCTreeR (supplementary information S7, Supplementary Material online).

### Stochastic Mapping of Multicellularity

Cytomorphology of species in this analysis was established from the published literature (supplementary information S4, Supplementary Material online). Previous analyses have coded characters for chlorophytes (Del Cortona et al. 2020; Li et al. 2021) which were transferred to this study. The tree topology was fixed based on the focal maximum likelihood analysis (Fig. 1) whilst branch lengths correspond to the timescale analysis presented in Fig. 3. Traits were discretely characterized as unicellular, filamentous, colony, siphonocladous, siphonous, sarcinoid, and multicellular. Ancestral states were estimated in R (R Core Team 2023) using the package phytools (Revell 2012). Briefly, likelihood ancestral states for discretely valued traits (e.g. multicellularity) were estimated using a continuous-time Markov chain model (Revell 2012). The MCMC approach is used to sample character histories from their posterior probability distribution (Huelsenbeck et al. 2003). To sample a greater portion of the character history, 1,000 stochastic maps were produced and summarized (supplementary Information S4, Supplementary Material online).

As multicellularity can be classified in different ways, various classifications of multicellularity were tested (supplementary Informations S4 and S5, Supplementary Material online). These included coding filamentous organisms as a separate classification to multicellular organisms (supplementary information S5a, Supplementary Material online), incorporating filamentous within multicellularity (supplementary information S5b, Supplementary Material online) and coding multicellularity as a binary character e.g. unicellular or multicellular (supplementary information S5c, Supplementary Material online). Additionally for this binary definition of multicellularity, posterior probability density was calculated with the densityMap function from phytools (supplementary information S5d, Supplementary Material online). These analyses were largely congruent with each other. The ancestral states presented in Fig. 4 are summarized from supplementary information S5b, Supplementary Material online, based on the classification incorporating filamentous within multicellularity.

## Supplementary Material

evae026_Supplementary_Data

## Data Availability

All data are available in the main text, the Supplementary materials or available on Figshare: https://figshare.com/s/460c2a4704493e5bb3b9.
