# Supplementary material for: Cryogenian Origins of Multicellularity in Archaeplastida
Source: Genome Biol Evol. 2024 Feb 9;16(2):evae026. doi: 10.1093/gbe/evae026 (PMC10883732; doi:10.1093/gbe/evae026)
Supplement: evae026_Supplementary_Data [file evae026_supplementary_data.zip › SI GBE 2 - Copy.docx]

**Supplementary Information**

**Supplementary Information 1**

**
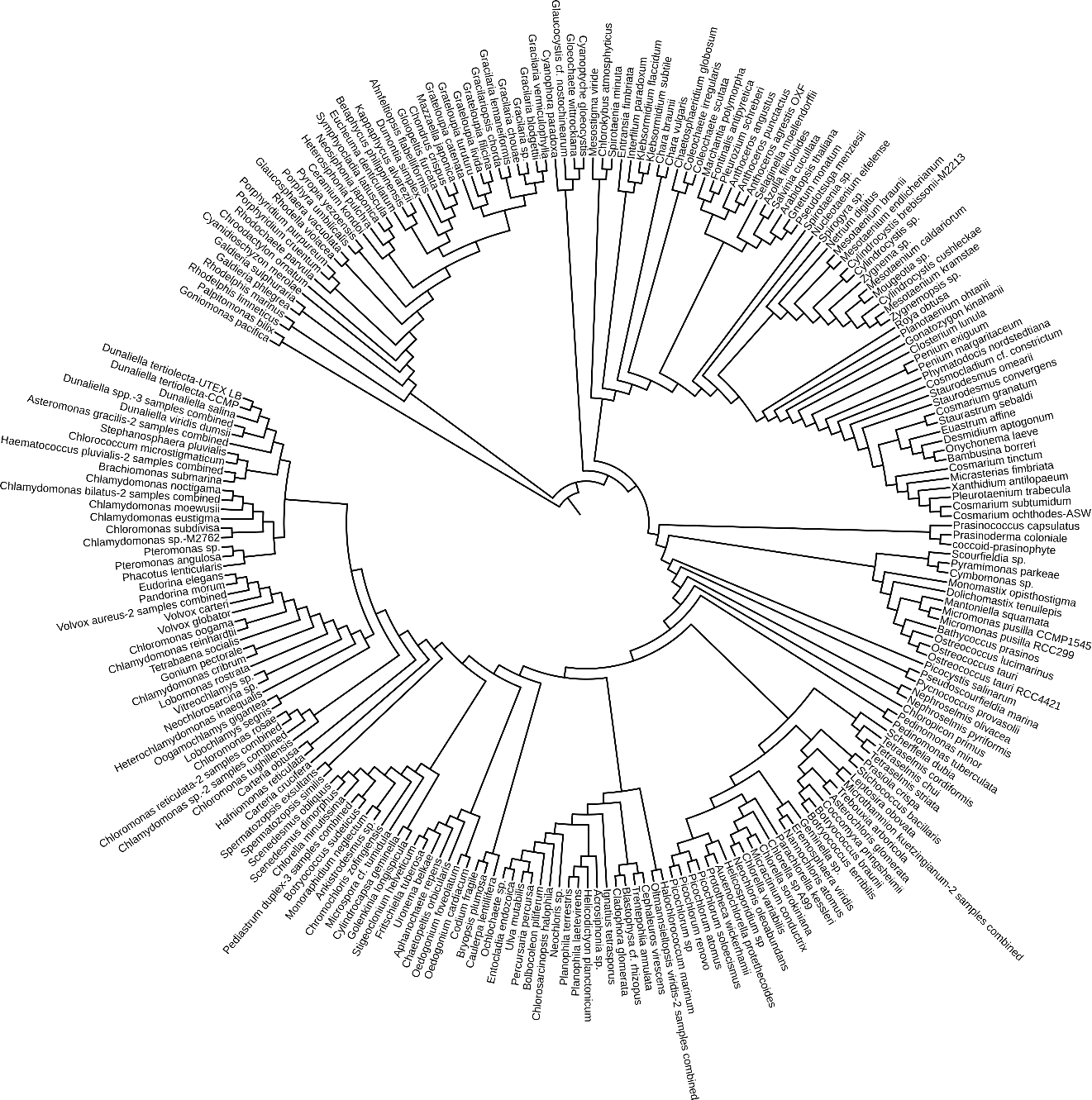
Supplementary Figure 1. Putative phylogeny of species used in this study.** Data used are sourced from genomes and the One Thousand Transcriptome Project [1–52].

**Supplementary Information 2**


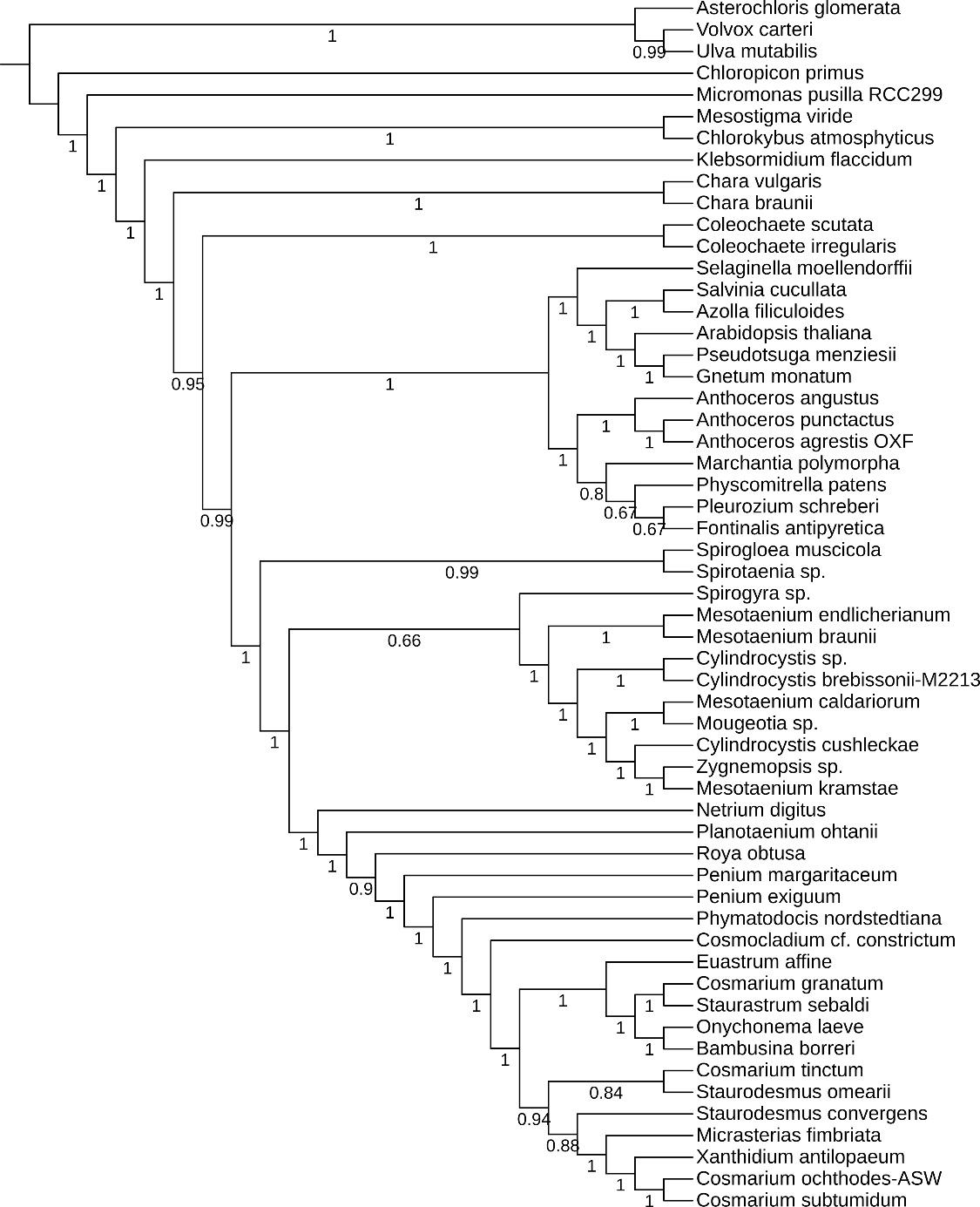


**Supplementary Figure 2a:** ASTRAL based species tree for Streptophyta data rich dataset

**
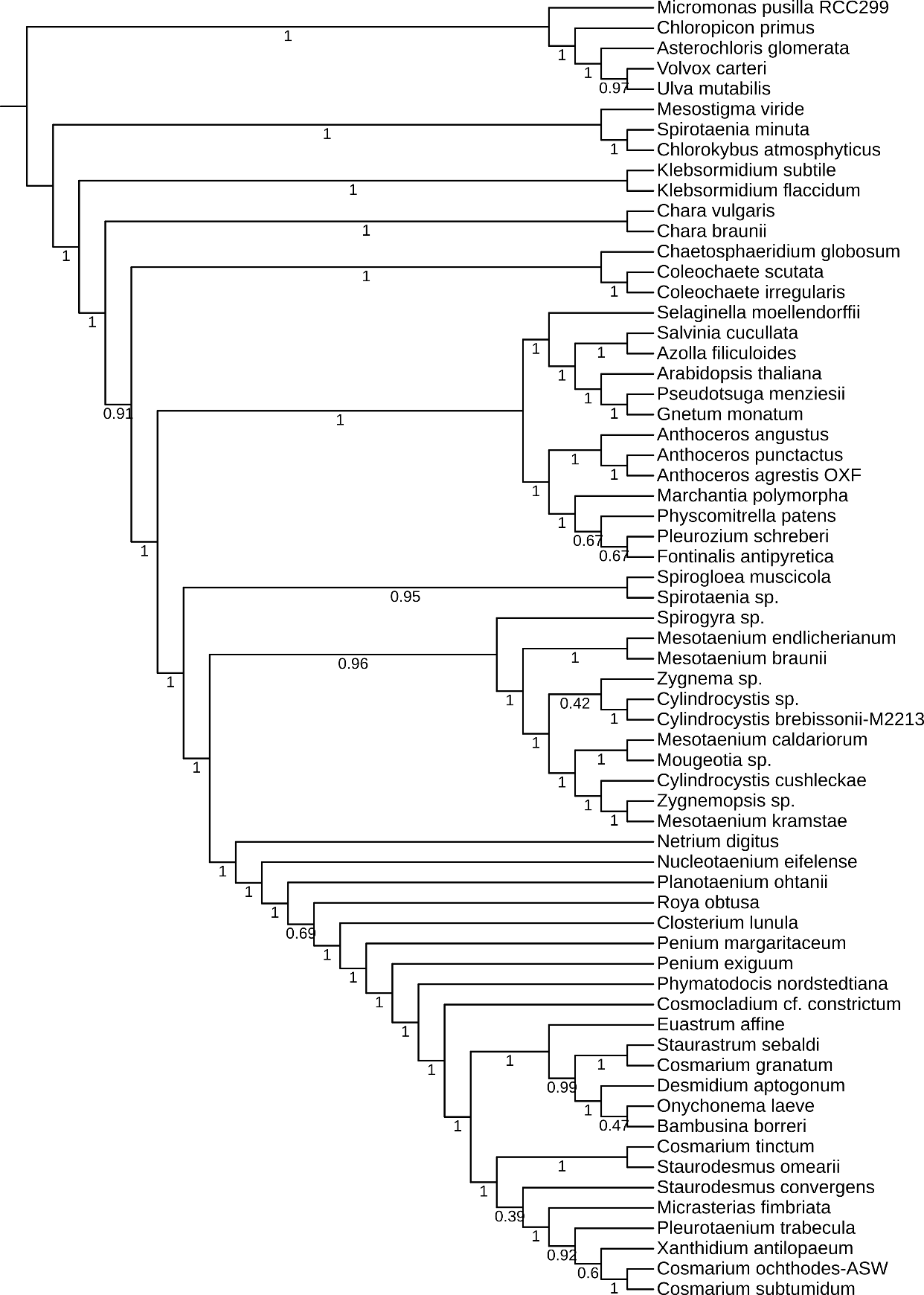
**

**Supplementary Figure 2b:** ASTRAL based species tree for Streptophyta taxon rich dataset


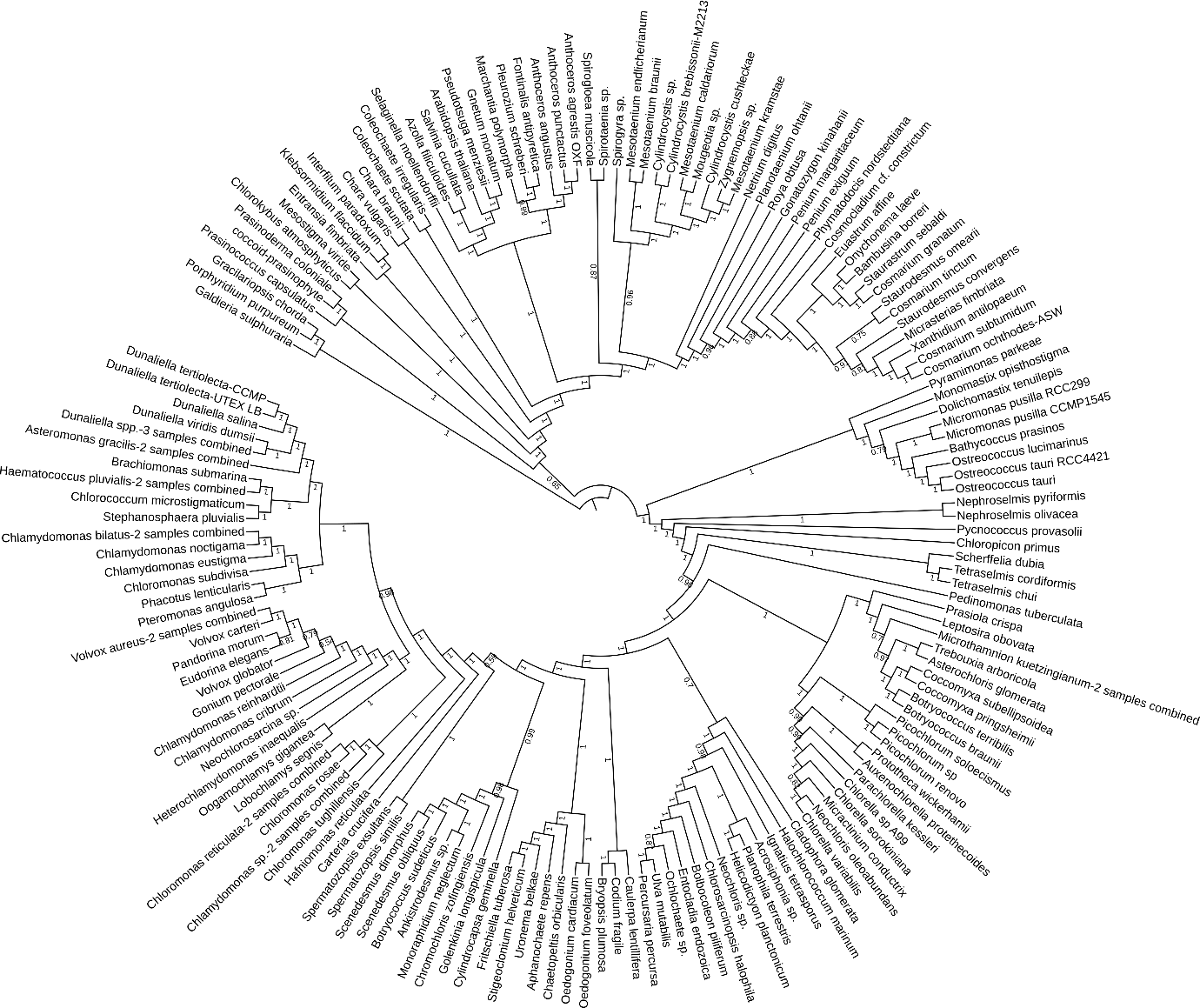


**Supplementary Figure 2c:** ASTRAL based species tree for Viridiplantae data rich dataset

**
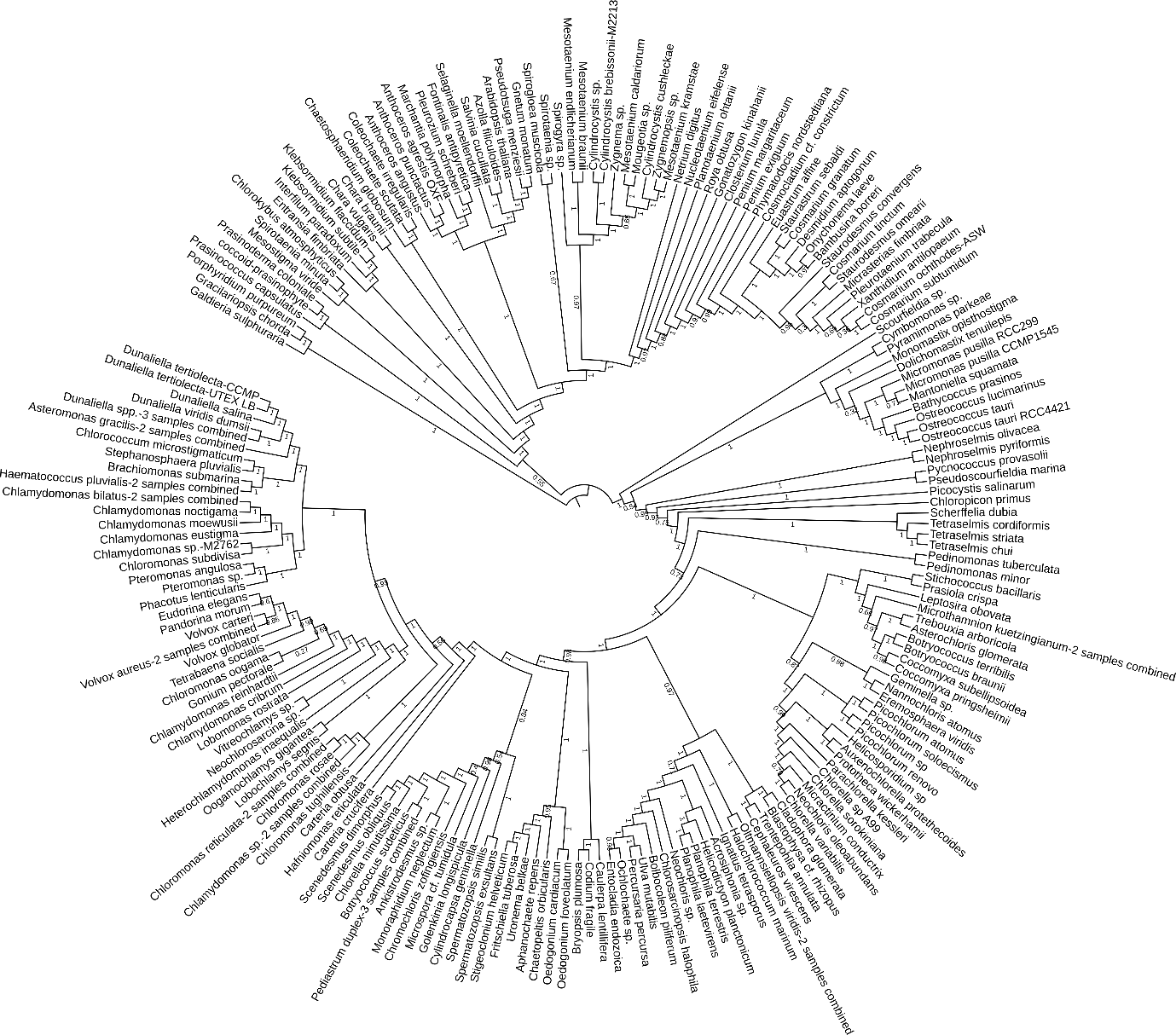
**

**Supplementary Figure 2d:** ASTRAL based species tree for Viridiplantae taxon rich dataset


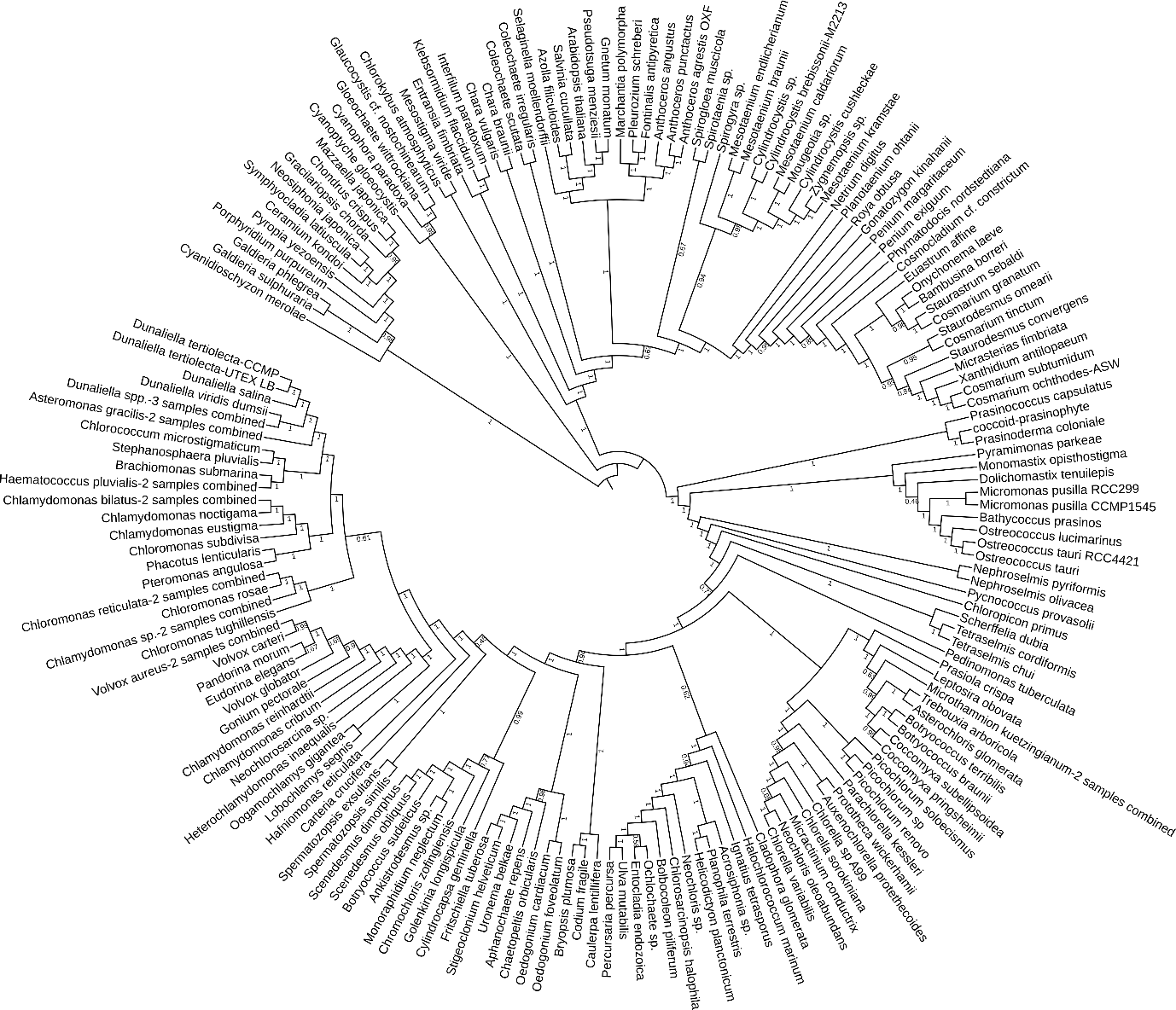


**Supplementary Figure 2e:** ASTRAL based species tree for Archaeplastida data rich dataset without outgroup taxa

**
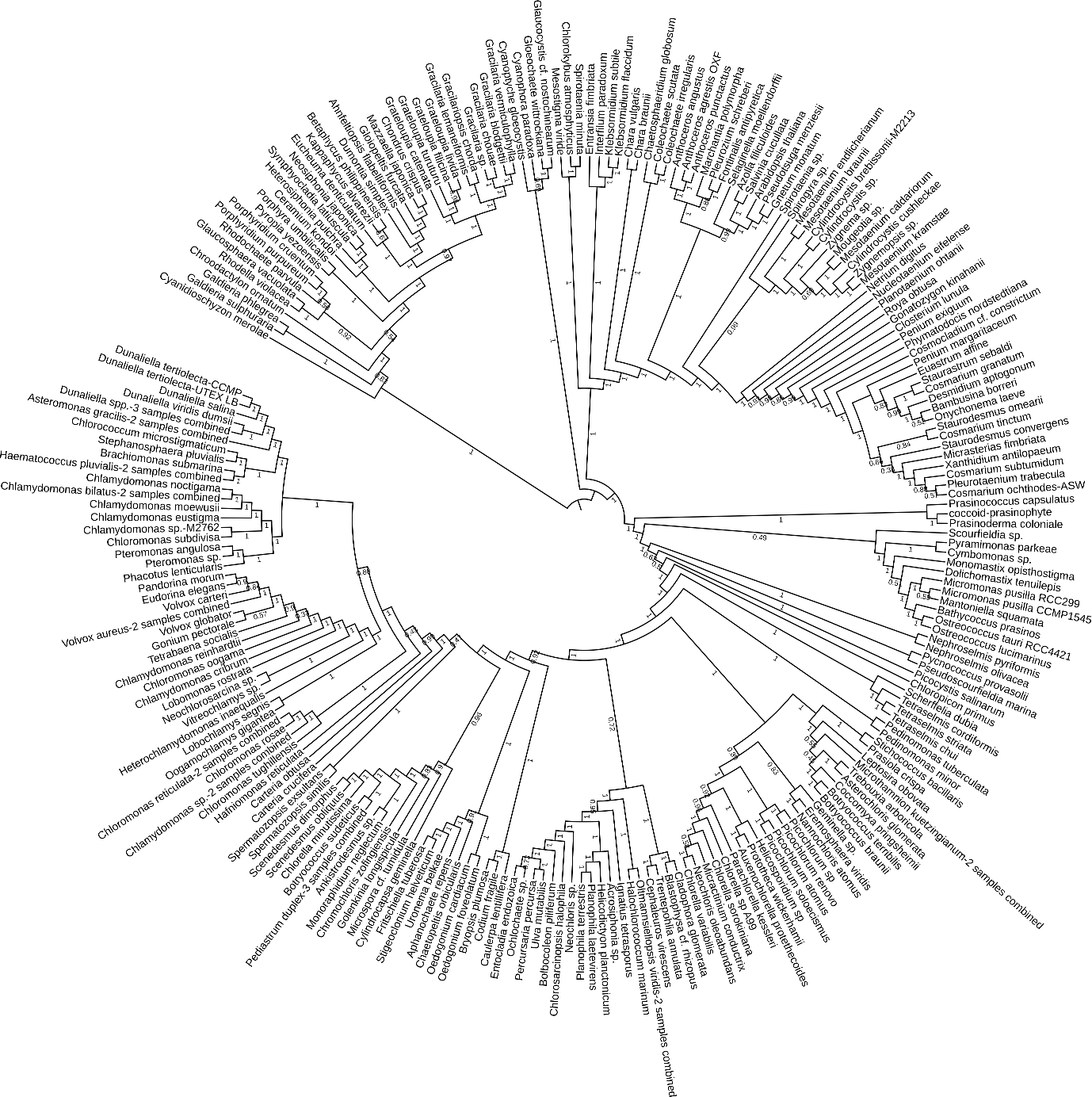
**

**Supplementary Figure 2f:** ASTRAL based species tree for Archaeplastida taxon rich dataset without outgroup taxa


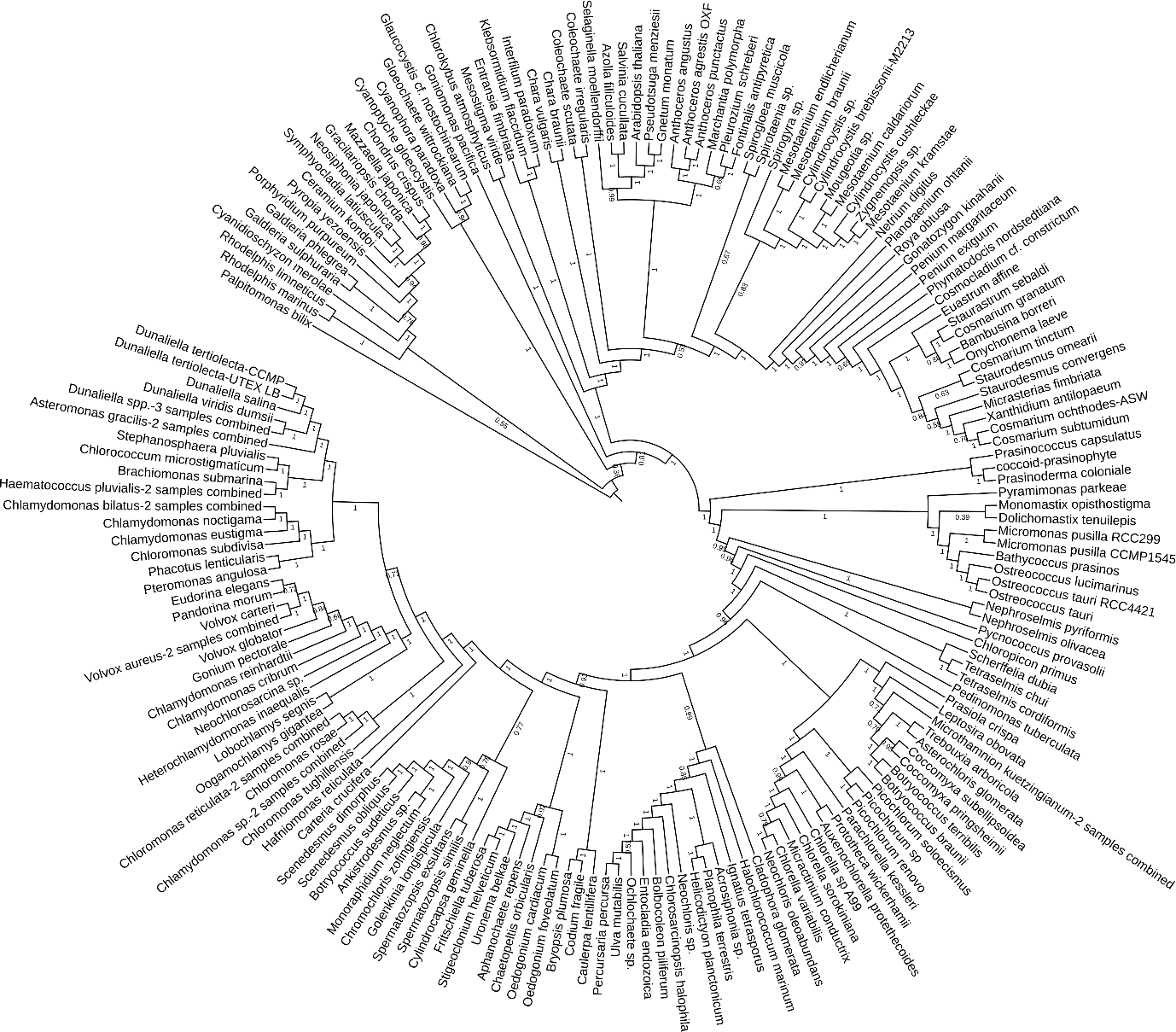


**Supplementary Figure 2g:** ASTRAL based species tree for Archaeplastida data rich dataset

**
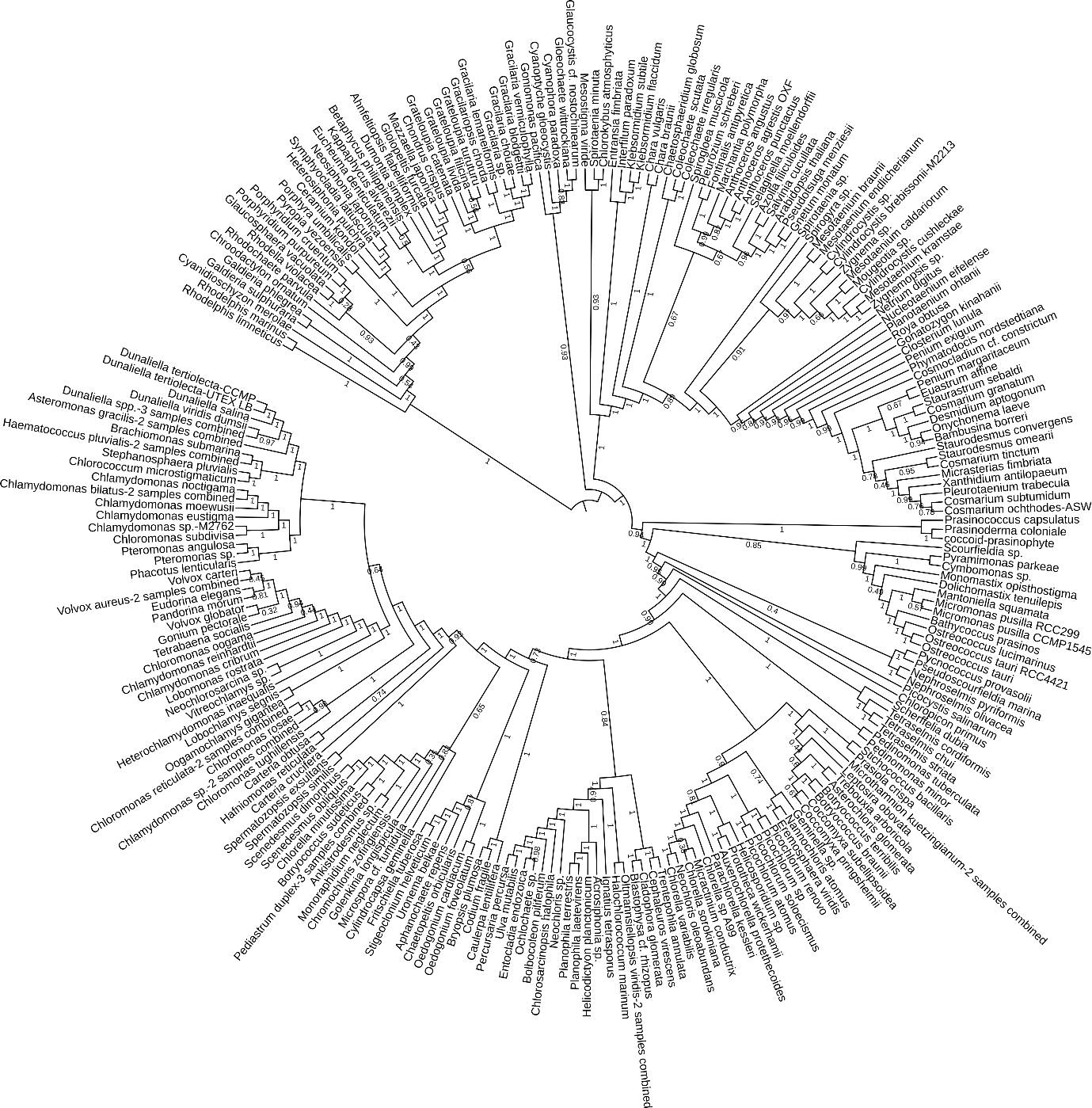
**

**Supplementary Figure 2h:** ASTRAL based species tree for Archaeplastida taxon rich dataset

**Supplementary Information 3**

**Fossil calibrations for Molecular Clock Analysis**

**Calibration_1:** Crown Group Eukaryota

**Specimen and fossil taxon:** *Bangiomorpha pubescens*. (Holotype) HUPC 62912, Slide HUST-1A, England Finder coordinates: O-35.

Locality and Stratigraphy level: Lower Hunting Formation, Somerset Island, Arctic Canada. **Soft Minimum age:** 1030 Ma (1047 Ma +13/–17 Myr [53])

**Soft maximum age:** 1879.6 Ma

Age justification:

**Hard maximum**: is based on the maximum age interpretation of the Gunflint Chert, a diverse and well-documented microbiota that is widely interpreted to be absent of eukaryotes. This has been dated to 1878.3 Ma ± 1.3 Myr [54], yielding a maximum age of 1879.6 Ma.

**Calibration_2:** Archaeplastida: Rhodophyta – [Glaucophyta-Viridiplantae]

**Fossil taxon and specimen:** *Bangiomorpha pubescens*

***Phylogenetic justification:*** Morphological similarity of *Bangiomorpha pubescens* with modern red algae *Bangia* [55].
***Minimum age:*** 1030 Ma.
**Soft maximum age:** 1879.6 Ma

***Age justification:*** Re-Os isotopic aging of sedimentary rocks in the stratigraphic region in which *Bangiomorpha pubescens* was sampled date this fossil at 1.047 +0.013/–0.017 Ga. Therefore the minimum age is 1030 Ma [53]. **Hard maximum**: Based on the maximum age interpretation of the Gunflint Chert, a diverse and well-documented microbiota that is widely interpreted to be absent of eukaryotes. This has been dated to 1878.3 Ma ± 1.3 Myr [54], yielding a maximum age of 1879.6 Ma.

**Calibration_3:** crown Rhodophyta

**Fossil taxon and specimen:** *Thallophyca ramosa*

***Phylogenetic justification:*** A diverse assemblage of multicellular microscopic remains from the Ediacaran Weng’an Biota have been attributed to clades within crown-Rhodophyta [56]. While their anatomy is compatible with a rhodophyte affinity, they are missing key characters of the crown clade and, further, much of the diversity can be rationalised as developmental variation in a single taxon which Landon (2021) reduces through synonymy to *Thallophyca ramosa*. In an absence of further constraint, *Thallophyca ramose* is attributed to the rhodophyte total group.

Morphological similarity of *Bangiomorpha pubescens* with modern red algae *Bangia* [55].
***Minimum age:*** 574 Ma.
***Soft maximum age:*** 1879.6 Ma.
***Age justification:*** Yang et al [57] constrain the age of the Weng’an Biota to precede the Shuram anomaly, the base of which they date to 574 Ma.

**Calibration_4:** Viridiplantae/Chloroplastida: Chlorophyta - Streptophyta

**Fossil taxon and specimen:** *Proterocladus antiquus* [58]

***Phylogenetic justification:*** Following Harris *et al.* [59]
***Minimum age:*** 940.4 Ma.
***Soft maximum age:*** 1879.6 Ma.
***Age justification:*** Following Harris *et al.* [59]

**Calibration_5:** Chlorophyta

**Fossil taxon and specimen:** *Proterocladus antiquus* [58]

***Phylogenetic justification:*** Following Harris *et al.* [59]
***Minimum age:*** 940.4 Ma.
***Soft maximum age:*** 1879.6 Ma.
***Age justification:*** Following Harris *et al.* [59]

**Calibration_6:** Chlorophyceae – Ulvophyceae

***Fossil taxon and specimen*:** *Palaeocymopolia silurica* [60]

***Phylogenetic justification:*** Following Morris et al [61]
***Minimum age:*** 438.3 Ma.

***Soft maximum age:*** 1879.6 Ma.
***Age justification:*** Following Morris et al [61]

**Calibration_7:** Trebouxiophyceae

**Fossil taxon and specimen:** *Botryococcus sp (previously Gloeocapsomorpha sp)* from the Upper Permian Hyland Bay Formation, Western Australia [62].

***Phylogenetic justification:*** Extant *Botryoccocus* are planktonic colonial algae found in brackish and fresh water. Their outer cell walls are formed of an acid-resistant biopolymer. Colonies of fossilised *Botryococcus* consist of cell clusters, with individual cells being oblong [62].

***Minimum age:*** 298.75 Ma.
***Soft maximum age:*** 1879.6 Ma Ma.
***Age justification:*** Botryococcus appears early within the fossil record, dating from the Precambrian (>542Ma) [63–66]. They are the largest contribution to the formation of crude oil and oil shales. In oil shales botryococcanes are present, which are lipids that co-localise with fossil Botryococcus [67].

**Calibration_8:** Streptophyta: Charophyta – Embryophyta

***Fossil taxon and specimen*:** *Tetrahedraletes* cf. *Medinensis* [68].

***Phylogenetic justification:*** Following Morris *et al.* [61]
***Minimum age:*** 469 Ma.
***Soft maximum age:*** 1879.6 Ma Ma.
***Age justification:*** Following Morris *et al.* [61]

**Calibration_9**: Embryophyta: Bryophyta – Tracheophyta

***Fossil taxon and specimen:*** *Tetrahedraletes cf. medinensis*

***Phylogenetic justification:*** Following Morris *et al.* [61]

***Minimum age:*** 469 Ma

***Maximum age:*** 515.5 Ma

***Age justification:*** Following Morris *et al.* [61]

**Calibration_10:** Anthocerotophyta

***Fossil taxon and specimen:*** Anthoceros sp.

***Phylogenetic justification:*** Following Harris *et al.* [59]

***Minimum age:*** 116.59 Ma

***Maximum age:*** 515 Ma

***Age justification:*** Following Harris *et al.* [59]

**Calibration_11:** Bryophyta

***Fossil taxon and specimen:*** Sphagnales

***Phylogenetic justification:*** Following Morris et al. [61]

***Minimum age:*** 330.7 Ma

***Maximum age:*** 515.5 Ma

***Age justification:*** Following Morris et al. [61]

**Calibration_12**: Tracheophyta: Lycopodiophyta – Euphyllophyta

***Fossil taxon and specimen:*** Zosterophyllum sp. and Chelinospora? sp.

***Phylogenetic justification:*** Following Harris *et al.* [59]

***Minimum age:*** 420.7 Ma

***Maximum age:*** 458.88 Ma

***Age justification:*** Following Harris *et al.* [59]

**Calibration_13**: Euphyllophyta

***Fossil taxon and specimen:*** *Kenrickia bivena*

***Phylogenetic justification:*** Following Harris *et al.* [59]

***Minimum age:*** 393.2 Ma

***Maximum age:*** 451 Ma

***Age justification:*** Following Harris *et al.* [59]

**Calibration_14**: Spermatophyta: Acrogymnospermae – Angiospermae

***Fossil taxon and specimen:*** *Cordaixylon iowensis*

***Phylogenetic justification:*** Following Morris *et al.* [61]

***Minimum age:*** 308.14 Ma

***Maximum age:*** 365.63 Ma

***Age justification:*** Following Morris *et al.* [61]

**Calibration_15**: Acrogymnospermae

***Fossil taxon and specimen:*** *Cordaixylon iowensis*

***Phylogenetic justification:*** Following Morris *et al.* [61]

***Minimum age:*** 308.14 Ma

***Maximum age:*** 365.63 Ma

***Age justification:*** Following Morris *et al.* [61]

**Supplementary Information 4**

Cytomorphological traits used in stochastic mapping alongside sources

| **Species** | **Character state** | **Character state (Filamentous as multicellular)** | **Ref.** |
| --- | --- | --- | --- |
| Goniomonas_pacifica | Unicellular | Unicellular | [69] |
| Palpitomonas_bilix | Unicellular | Unicellular | [69] |
| Ceramium_kondoi | Multicellular | Multicellular | [70] |
| Neosiphonia_japonica | Multicellular | Multicellular | [71] |
| Symphyocladia_latiuscula | Multicellular | Multicellular | [72] |
| Heterosiphonia_pulchra | Multicellular | Multicellular | [73] |
| Chondrus_crispus | Multicellular | Multicellular | [74] |
| Mazzaella_japonica | Multicellular | Multicellular | [75] |
| Gloiopeltis_furcata | Multicellular | Multicellular | [76] |
| Ahnfeltiopsis_flabelliformis | Multicellular | Multicellular | [77] |
| Dumontia_simplex | Multicellular | Multicellular | [78] |
| Eucheuma_denticulatum | Multicellular | Multicellular | [79] |
| Kappaphycus_alvarezii | Multicellular | Multicellular | [80] |
| Betaphycus_philippinensis | Multicellular | Multicellular | [81] |
| Gracilaria_blodgettii | Multicellular | Multicellular | [82] |
| Gracilaria_vermiculophylla | Multicellular | Multicellular | [82] |
| Gracilaria_chouae | Multicellular | Multicellular | [82] |
| Gracilaria_sp. | Multicellular | Multicellular | [82] |
| Gracilariopsis_chorda | Multicellular | Multicellular | [52] |
| Gracilaria_lemaneiformis | Multicellular | Multicellular | [82] |
| Grateloupia_catenata | Multicellular | Multicellular | [83] |
| Grateloupia_livida | Multicellular | Multicellular | [83] |
| Grateloupia_filicina | Multicellular | Multicellular | [83] |
| Grateloupia_turuturu | Multicellular | Multicellular | [83] |
| Porphyra_umbilicalis | Multicellular | Multicellular | [50] |
| Pyropia_yezoensis | Multicellular | Multicellular | [84] |
| Porphyridium_cruentum | Unicellular | Unicellular | [7] |
| Porphyridium_purpureum | Unicellular | Unicellular | [7] |
| Rhodella_violacea | Unicellular | Unicellular | [85] |
| Glaucosphaera_vacuolata | Unicellular | Unicellular | [86] |
| Rhodochaete_parvula | Filamentous | Multicellular | [87] |
| Chroodactylon_ornatum | Filamentous | Multicellular | [88] |
| Galdieria_phlegrea | Unicellular | Unicellular | [89] |
| Galdieria_sulphuraria | Unicellular | Unicellular | [89] |
| Cyanidioschyzon_merolae | Unicellular | Unicellular | [89] |
| Cyanophora_paradoxa | Unicellular | Unicellular | [90] |
| Glaucocystis_cf._nostochinearum | Unicellular | Unicellular | [90] |
| Gloeochaete_wittrockiana | Unicellular | Unicellular | [90] |
| Cyanoptyche_gloeocystis | Unicellular | Unicellular | [90] |
| Prasinococcus_capsulatus | Unicellular | Unicellular | [91] |
| Prasinoderma_coloniale | Unicellular | Unicellular | [92] |
| coccoid_prasinophyte | Unicellular | Unicellular | [14] |
| Scourfieldia_sp. | Unicellular | Unicellular | [14] |
| Bathycoccus_prasinos | Unicellular | Unicellular | [14] |
| Ostreococcus_lucimarinus | Unicellular | Unicellular | [14] |
| Ostreococcus_tauri | Unicellular | Unicellular | [14] |
| Ostreococcus_tauri_RCC4421 | Unicellular | Unicellular | [14] |
| Mantoniella_squamata | Unicellular | Unicellular | [93] |
| Micromonas_pusilla_CCMP1545 | Unicellular | Unicellular | [14] |
| Micromonas_pusilla_RCC299 | Unicellular | Unicellular | [14] |
| Dolichomastix_tenuilepis | Unicellular | Unicellular | [14] |
| Monomastix_opisthostigma | Unicellular | Unicellular | [14] |
| Pyramimonas_parkeae | Unicellular | Unicellular | [14] |
| Cymbomonas_sp. | Unicellular | Unicellular | [14] |
| Pseudoscourfieldia_marina | Unicellular | Unicellular | [14] |
| Pycnococcus_provasolii | Unicellular | Unicellular | [14] |
| Nephroselmis_olivacea | Unicellular | Unicellular | [14] |
| Nephroselmis_pyriformis | Unicellular | Unicellular | [14] |
| Picocystis_salinarum | Unicellular | Unicellular | [14] |
| Picocystis_ML. | Unicellular | Unicellular | [14] |
| Chloropicon_primus | Unicellular | Unicellular | [14] |
| Chloroidium_sp. | Unicellular | Unicellular | [94] |
| Pedinomonas_minor | Unicellular | Unicellular | [95] |
| Pedinomonas_tuberculata | Unicellular | Unicellular | [95] |
| Scherffelia_dubia | Unicellular | Unicellular | [96] |
| Tetraselmis_chui | Unicellular | Unicellular | [96] |
| Tetraselmis_striata | Unicellular | Unicellular | [96] |
| Tetraselmis_cordiformis | Unicellular | Unicellular | [96] |
| Asterochloris_glomerata | Unicellular | Unicellular | [15] |
| Trebouxia_arboricola | Unicellular | Unicellular | [96] |
| Botryococcus_braunii | Colony | Colony | [96] |
| Botryococcus_terribilis | Colony | Colony | [96] |
| Coccomyxa_pringsheimii | Unicellular | Unicellular | [19] |
| Coccomyxa_subellipsoidea | Unicellular | Unicellular | [19] |
| Leptosira_obovata | Multicellular | Multicellular | [97] |
| Microthamnion_kuetzingianum_2_samples_combined | Filamentous | Multicellular | [98] |
| Prasiola_crispa | Multicellular | Multicellular | [99] |
| Stichococcus_bacillaris | Unicellular | Unicellular | [100] |
| Auxenochlorella_protethecoides | Unicellular | Unicellular | [16] |
| Prototheca_wickerhamii | Unicellular | Unicellular | [101] |
| Helicosporidium_sp | Filamentous | Multicellular | [102] |
| Picochlorum_renovo | Unicellular | Unicellular | [103] |
| Picochlorum_sp | Unicellular | Unicellular | [103] |
| Picochlorum_soloecismus | Unicellular | Unicellular | [103] |
| Picochlorum_atomus | Unicellular | Unicellular | [103] |
| Chlorella_sorokiniana | Unicellular | Unicellular | [103] |
| Micractinium_conductrix | Unicellular | Unicellular | [21] |
| Chlorella_variabilis | Unicellular | Unicellular | [104] |
| Neochloris_oleoabundans | Unicellular | Unicellular | [105] |
| Chlorella_sp_A99 | Unicellular | Unicellular | [96] |
| Parachlorella_kessleri | Unicellular | Unicellular | [106] |
| Eremosphaera_viridis | Unicellular | Unicellular | [106] |
| Nannochloris_atomus | Unicellular | Unicellular | [96] |
| Geminella_sp. | Unicellular | Unicellular | [106] |
| Blastophysa_cf._rhizopus | Siphonocladous | Siphonocladous | [96] |
| Cladophora_glomerata | Siphonocladous | Siphonocladous | [96] |
| Cephaleuros_virescens | Multicellular | Multicellular | [96] |
| Trentepohlia_annulata | Filamentous | Multicellular | [107] |
| Bolbocoleon_piliferum | Filamentous | Multicellular | [110] |
| Entocladia_endozoica | Filamentous | Multicellular | [108] |
| Ochlochaete_sp. | Filamentous | Multicellular | [111] |
| Percursaria_percursa | Filamentous | Multicellular | [112] |
| Ulva_mutabilis | Multicellular | Multicellular | [109] |
| Chlorosarcinopsis_halophila | Unicellular | Unicellular | [113] |
| Neochloris_sp. | Unicellular | Unicellular | [114] |
| Helicodictyon_planctonicum | Multicellular | Multicellular | [96] |
| Planophila_laetevirens | Unicellular | Unicellular | [96] |
| Planophila_terrestris | Unicellular | Unicellular | [96] |
| Acrosiphonia_sp. | Siphonocladous | Siphonocladous | [96] |
| Ignatius_tetrasporus | Colony | Colony | [96] |
| Halochlorococcum_marinum | Unicellular | Unicellular | [96] |
| Oltmannsiellopsis_viridis_2_samples_combined | Unicellular | Unicellular | [96] |
| Bryopsis_plumosa | Siphonous | Siphonous | [96] |
| Codium_fragile | Siphonous | Siphonous | [96] |
| Caulerpa_lentillifera | Siphonous | Siphonous | [96] |
| Chaetopeltis_orbicularis | Filamentous | Multicellular | [115] |
| Fritschiella_tuberosa | Filamentous | Multicellular | [116] |
| Stigeoclonium_helveticum | Filamentous | Multicellular | [117] |
| Uronema_belkae | Filamentous | Multicellular | [118] |
| Aphanochaete_repens | Multicellular | Multicellular | [96] |
| Oedogonium_cardiacum | Multicellular | Multicellular | [96] |
| Oedogonium_foveolatum | Multicellular | Multicellular | [96] |
| Asteromonas_gracilis_2_samples_combined | Unicellular | Unicellular | [106] |
| Dunaliella_salina | Unicellular | Unicellular | [96] |
| Dunaliella_tertiolecta_CCMP | Unicellular | Unicellular | [96] |
| Dunaliella_tertiolecta_UTEX_LB | Unicellular | Unicellular | [96] |
| Dunaliella_viridis_dumsii | Unicellular | Unicellular | [96] |
| Dunaliella_spp._3_samples_combined | Unicellular | Unicellular | [96] |
| Brachiomonas_submarina | Unicellular | Unicellular | [119] |
| Haematococcus_pluvialis_2_samples_combined | Unicellular | Unicellular | [96] |
| Chlorococcum_microstigmaticum | Unicellular | Unicellular | [120] |
| Stephanosphaera_pluvialis | Colony | Colony | [121] |
| Chlamydomonas_bilatus_2_samples_combined | Unicellular | Unicellular | [122] |
| Chlamydomonas_noctigama | Unicellular | Unicellular | [122] |
| Chlamydomonas_moewusii | Unicellular | Unicellular | [122] |
| Chlamydomonas_eustigma | Unicellular | Unicellular | [122] |
| Chlamydomonas_sp._M2762 | Unicellular | Unicellular | [122] |
| Chloromonas_subdivisa | Unicellular | Unicellular | [122] |
| Phacotus_lenticularis | Unicellular | Unicellular | [123] |
| Pteromonas_angulosa | Unicellular | Unicellular | [96] |
| Pteromonas_sp. | Unicellular | Unicellular | [96] |
| Chloromonas_reticulata_2_samples_combined | Unicellular | Unicellular | [122] |
| Chloromonas_rosae | Unicellular | Unicellular | [122] |
| Chloromonas_tughillensis | Unicellular | Unicellular | [122] |
| Chlamydomonas_sp._2_samples_combined | Unicellular | Unicellular | [122] |
| Chlamydomonas_reinhardtii | Unicellular | Unicellular | [96] |
| Chloromonas_oogama | Unicellular | Unicellular | [122] |
| Eudorina_elegans | Colony | Colony | [124] |
| Pandorina_morum | Colony | Colony | [124] |
| Volvox_carteri | Colony | Colony | [96] |
| Volvox_aureus_2_samples_combined | Colony | Colony | [96] |
| Volvox_globator | Colony | Colony | [96] |
| Gonium_pectorale | Colony | Colony | [106] |
| Tetrabaena_socialis | Colony | Colony | [124] |
| Chlamydomonas_cribrum | Unicellular | Unicellular | [124] |
| Lobomonas_rostrata | Unicellular | Unicellular | [125] |
| Neochlorosarcina_sp. | Sarcinoid | Sarcinoid | [126] |
| Vitreochlamys_sp. | Unicellular | Unicellular | [127] |
| Heterochlamydomonas_inaequalis | Unicellular | Unicellular | [131] |
| Lobochlamys_segnis | Unicellular | Unicellular | [128] |
| Oogamochlamys_gigantea | Unicellular | Unicellular | [129] |
| Carteria_obtusa | Unicellular | Unicellular | [106] |
| Hafniomonas_reticulata | Unicellular | Unicellular | [130] |
| Carteria_crucifera | Unicellular | Unicellular | [96] |
| Cylindrocapsa_geminella | Filamentous | Multicellular | [132] |
| Golenkinia_longispicula | Unicellular | Unicellular | [96] |
| Spermatozopsis_similis | Unicellular | Unicellular | [96] |
| Spermatozopsis_exsultans | Unicellular | Unicellular | [96] |
| Microspora_cf._tumidula | Filamentous | Multicellular | [133] |
| Chromochloris_zofingiensis | Unicellular | Unicellular | [134] |
| Botryococcus_sudeticus | Colony | Colony | [135] |
| Pediastrum_duplex_3_samples_combined | Colony | Colony | [96] |
| Chlorella_minutissima | Unicellular | Unicellular | [104] |
| Scenedesmus_dimorphus | Colony | Colony | [96] |
| Scenedesmus_obliquus | Colony | Colony | [96] |
| Ankistrodesmus_sp. | Unicellular | Unicellular | [136] |
| Raphidocelis_subcaptita | Unicellular | Unicellular | [137] |
| Monoraphidium_neglectum | Unicellular | Unicellular | [138] |
| Mesostigma_viride | Unicellular | Unicellular | [29] |
| Chlorokybus_atmosphyticus | Sarcinoid | Sarcinoid | [29] |
| Spirotaenia_minuta | Unicellular | Unicellular | [139] |
| Entransia_fimbriata | Filamentous | Multicellular | [140] |
| Interfilum_paradoxum | Filamentous | Multicellular | [142] |
| Klebsormidium_flaccidum | Filamentous | Multicellular | [142] |
| Klebsormidium_subtile | Filamentous | Multicellular | [142] |
| Chara_braunii | Multicellular | Multicellular | [39] |
| Chara_vulgaris | Multicellular | Multicellular | [144] |
| Chaetosphaeridium_globosum | Sarcinoid | Sarcinoid |  |
| Coleochaete_irregularis | Sarcinoid | Sarcinoid |  |
| Coleochaete_scutata | Sarcinoid | Sarcinoid |  |
| Cosmarium_granatum | Unicellular | Unicellular | [141] |
| Staurastrum_sebaldi | Unicellular | Unicellular | [143] |
| Desmidium_aptogonum | Filamentous | Multicellular | [145] |
| Bambusina_borreri | Filamentous | Multicellular | [146] |
| Onychonema_laeve | Filamentous | Multicellular | [145] |
| Euastrum_affine | Unicellular | Unicellular | [147] |
| Cosmarium_subtumidum | Unicellular | Unicellular | [148] |
| Cosmarium_ochthodes_ASW | Unicellular | Unicellular | [148] |
| Pleurotaenium_trabecula | Unicellular | Unicellular | [149] |
| Xanthidium_antilopaeum | Unicellular | Unicellular |  |
| Micrasterias_fimbriata | Unicellular | Unicellular | [150] |
| Cosmarium_tinctum | Unicellular | Unicellular | [148] |
| Staurodesmus_omearii | Unicellular | Unicellular |  |
| Staurodesmus_convergens | Unicellular | Unicellular |  |
| Staurodesmus_convergens | Unicellular | Unicellular |  |
| Cosmocladium_cf._constrictum | Unicellular | Unicellular | [151] |
| Phymatodocis_nordstedtiana | Filamentous | Multicellular | [152] |
| Penium_exiguum | Unicellular | Unicellular | [153] |
| Penium_margaritaceum | Unicellular | Unicellular | [153] |
| Closterium_lunula | Unicellular | Unicellular | [154] |
| Gonatozygon_kinahanii | Filamentous | Multicellular | [146] |
| Roya_obtusa | Unicellular | Unicellular | [155] |
| Nucleotaenium_eifelense | Unicellular | Unicellular | [156] |
| Planotaenium_ohtanii | Unicellular | Unicellular | [156] |
| Netrium_digitus | Unicellular | Unicellular | [157] |
| Cylindrocystis_brebissonii_M2213 | Unicellular | Unicellular | [158] |
| Cylindrocystis_sp. | Unicellular | Unicellular | [158] |
| Cylindrocystis_cushleckae | Unicellular | Unicellular | [158] |
| Mesotaenium_kramstae | Unicellular | Unicellular | [159] |
| Zygnemopsis_sp. | Filamentous | Multicellular | [160] |
| Mesotaenium_caldariorum | Unicellular | Unicellular | [159] |
| Mougeotia_sp. | Filamentous | Multicellular | [161] |
| Zygnema_sp. | Filamentous | Multicellular | [143] |
| Mesotaenium_braunii | Unicellular | Unicellular | [155] |
| Mesotaenium_endlicherianum | Unicellular | Unicellular | [155] |
| Spirogyra_sp. | Filamentous | Multicellular | [162] |
| Spirotaenia_sp. | Unicellular | Unicellular | [139] |
| Spirogloea muscicola | Unicellular | Unicellular | [34] |
| Anthoceros_angustus | Multicellular | Multicellular | [163] |
| Anthoceros_punctactus | Multicellular | Multicellular | [163] |
| Anthoceros_agrestis_OXF | Multicellular | Multicellular | [163] |
| Fontinalis_antipyretica | Multicellular | Multicellular | [163] |
| Pleurozium_schreberi | Multicellular | Multicellular | [163] |
| Marchantia_polymorpha | Multicellular | Multicellular | [163] |
| Selaginella_moellendorffii | Multicellular | Multicellular | [163] |
| Azolla_filiculoides | Multicellular | Multicellular | [163] |
| Salvinia_cucullata | Multicellular | Multicellular | [163] |
| Gnetum_monatum | Multicellular | Multicellular | [163] |
| Pseudotsuga_menziesii | Multicellular | Multicellular | [163] |
| Arabidopsis_thaliana | Multicellular | Multicellular | [163] |

**Supplementary Information 5**

**
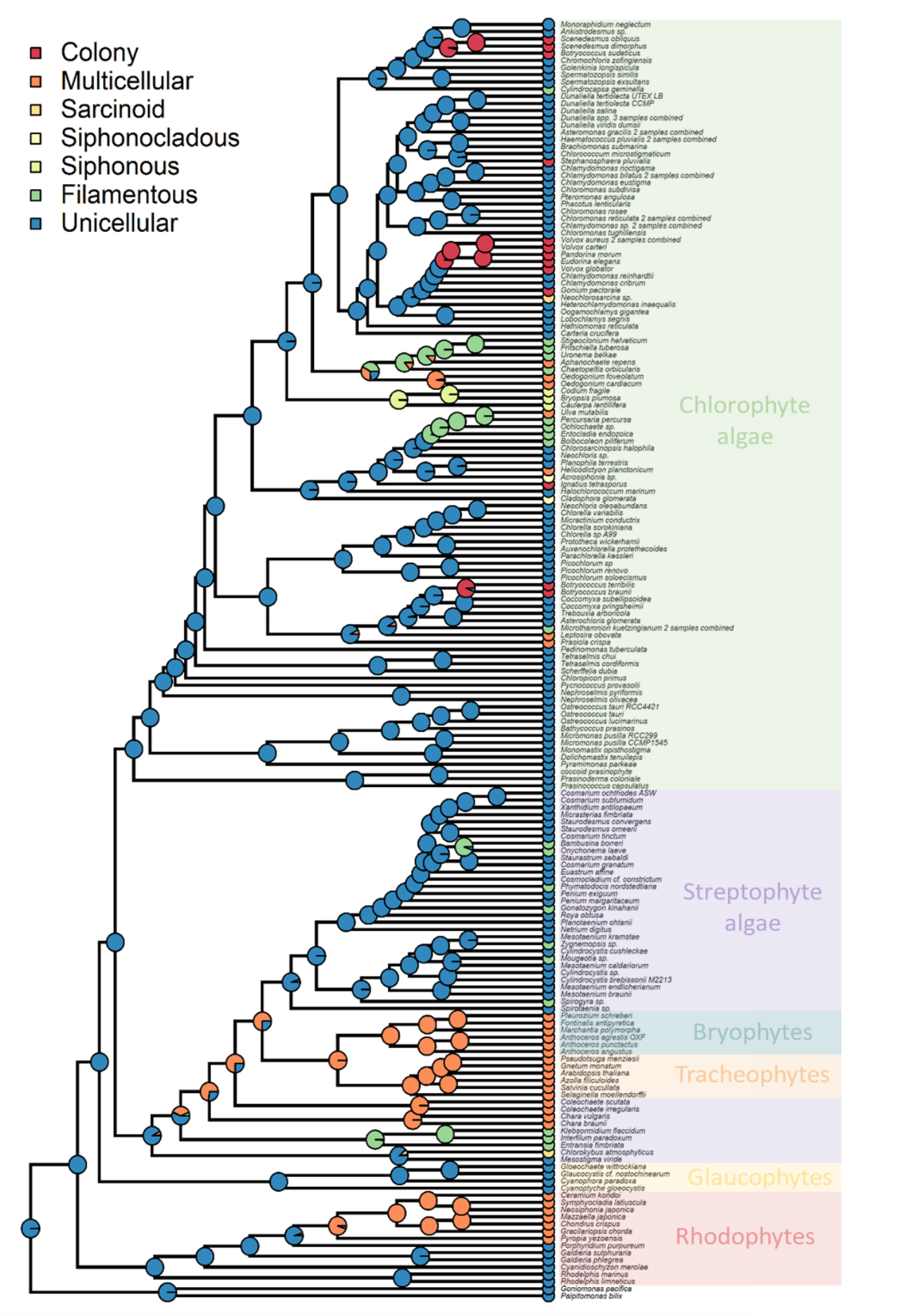
**Stochastic mapping of cytomorphological traits

**Supplementary Information 5a:** Summary of 1000 stochastic character maps of multicellularity including filamentous as a distinct character state. The tree is based on Figure 1 and branch lengths correspond to divergence times in Figure 3.

**
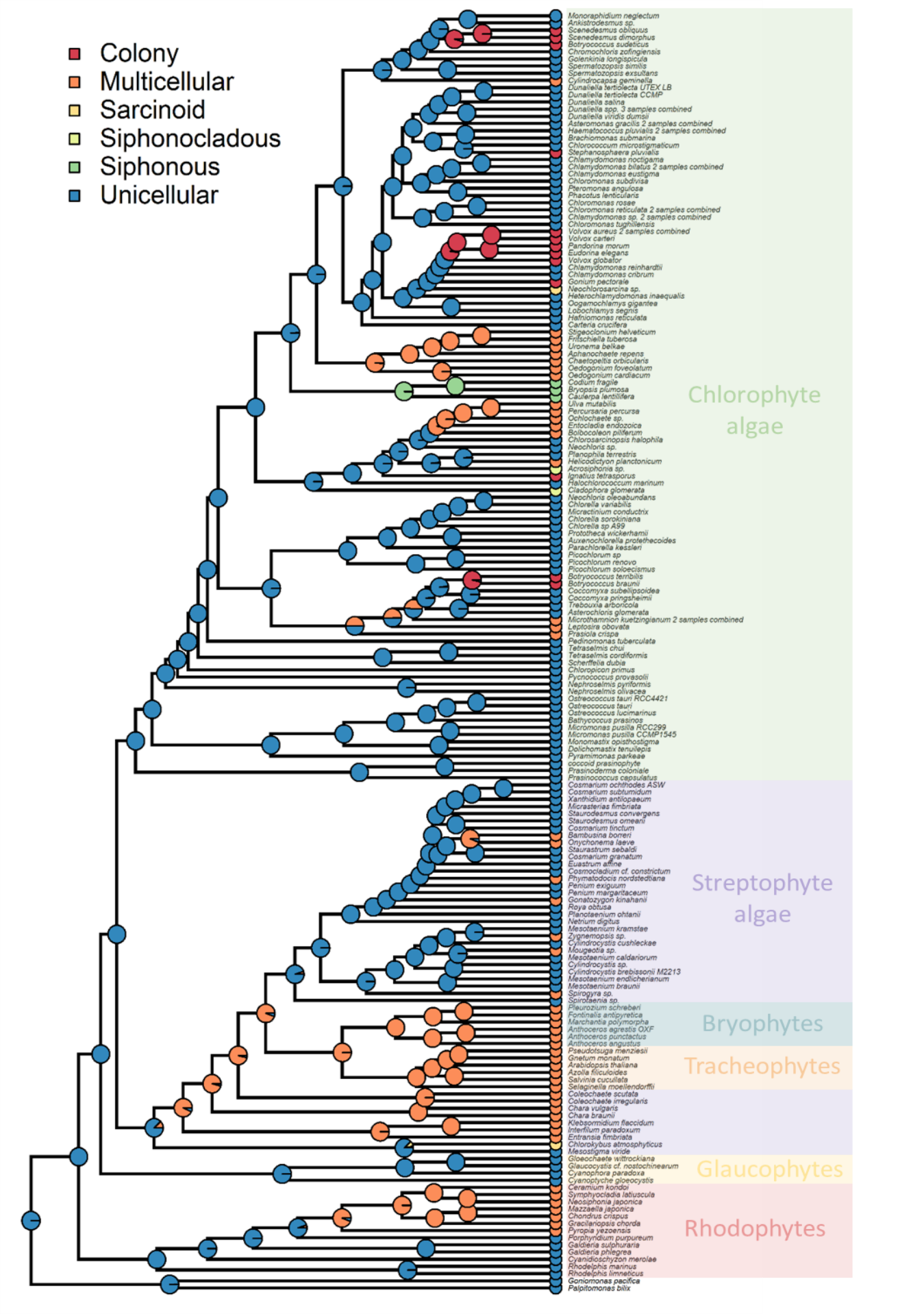
**

**Supplementary Information 5b:** Summary of 1000 stochastic character maps of multicellularity incorporating filamentous within multicellular character. The tree is based on Figure 1 and branch lengths correspond to divergence times in Figure 3.

**
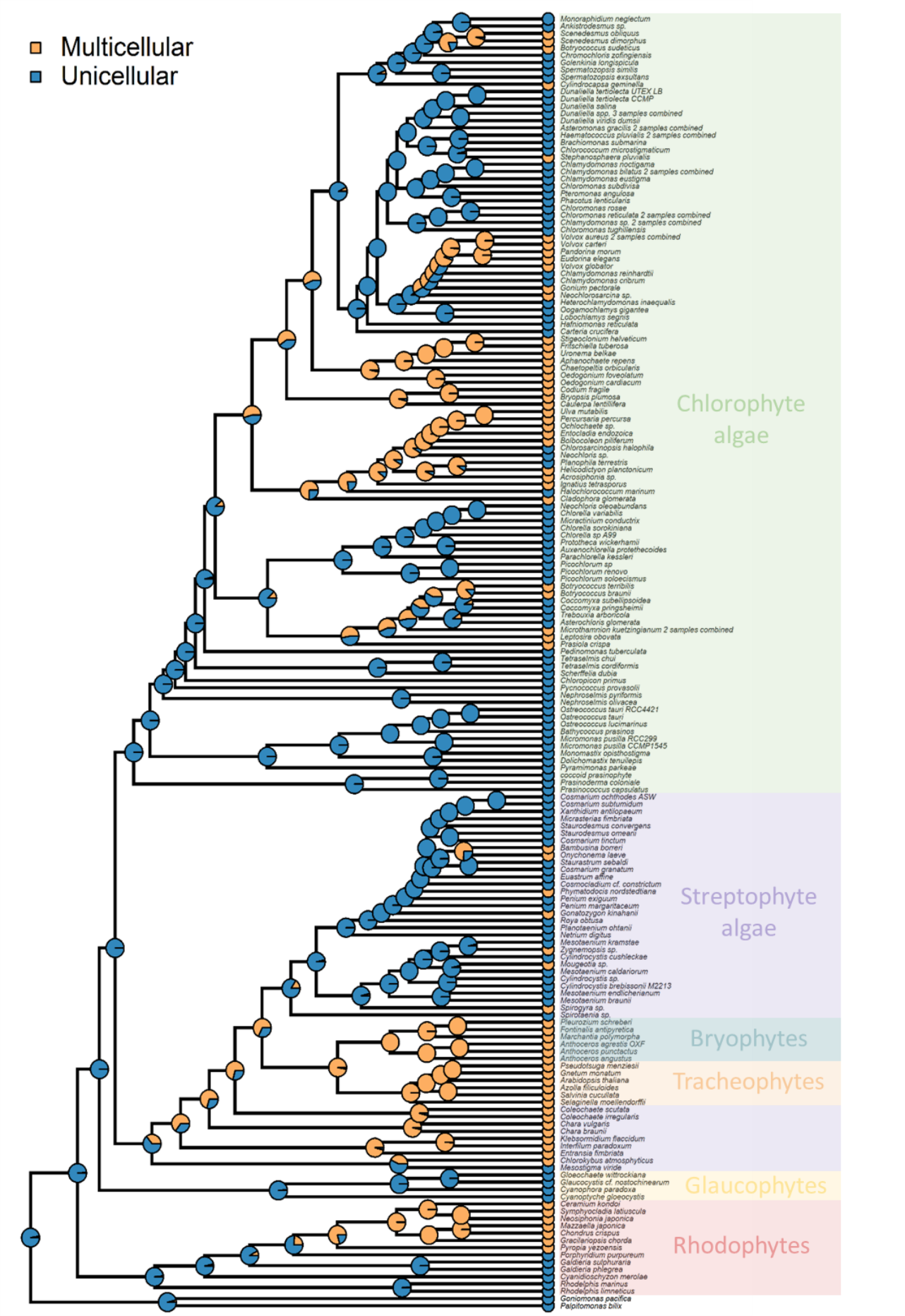
**

**Supplementary Information 5c:** Summary of 1000 stochastic character maps of multicellularity as a binary character: unicellular or multicellular. The tree is based on Figure 1 and branch lengths correspond to divergence times in Figure 3. The pie colour denotes the posterior probability of being multicellular (orange) or unicellular (blue).


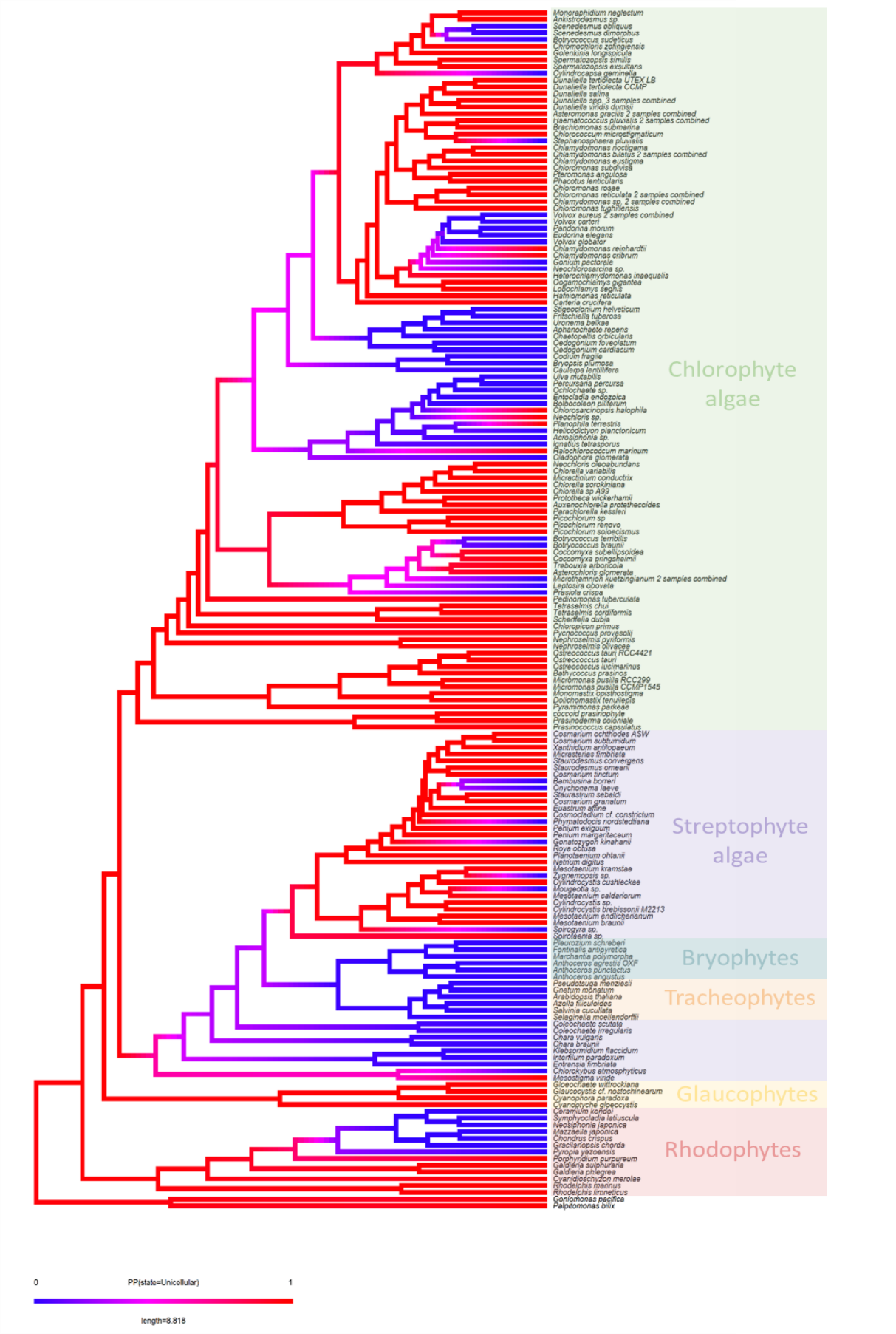


**Supplementary Information 5d:** Summary of 1000 stochastic character maps showing the posterior probability density of multicellular states as a binary character: unicellular or multicellular. The tree is based on Figure 1 and branch lengths correspond to divergence times in Figure 3. The colour of edges denotes the posterior probability of being multicellular or unicellular. Red indicates high posterior probability of being unicellular whilst blue indicates high posterior probability of being multicellular.

**
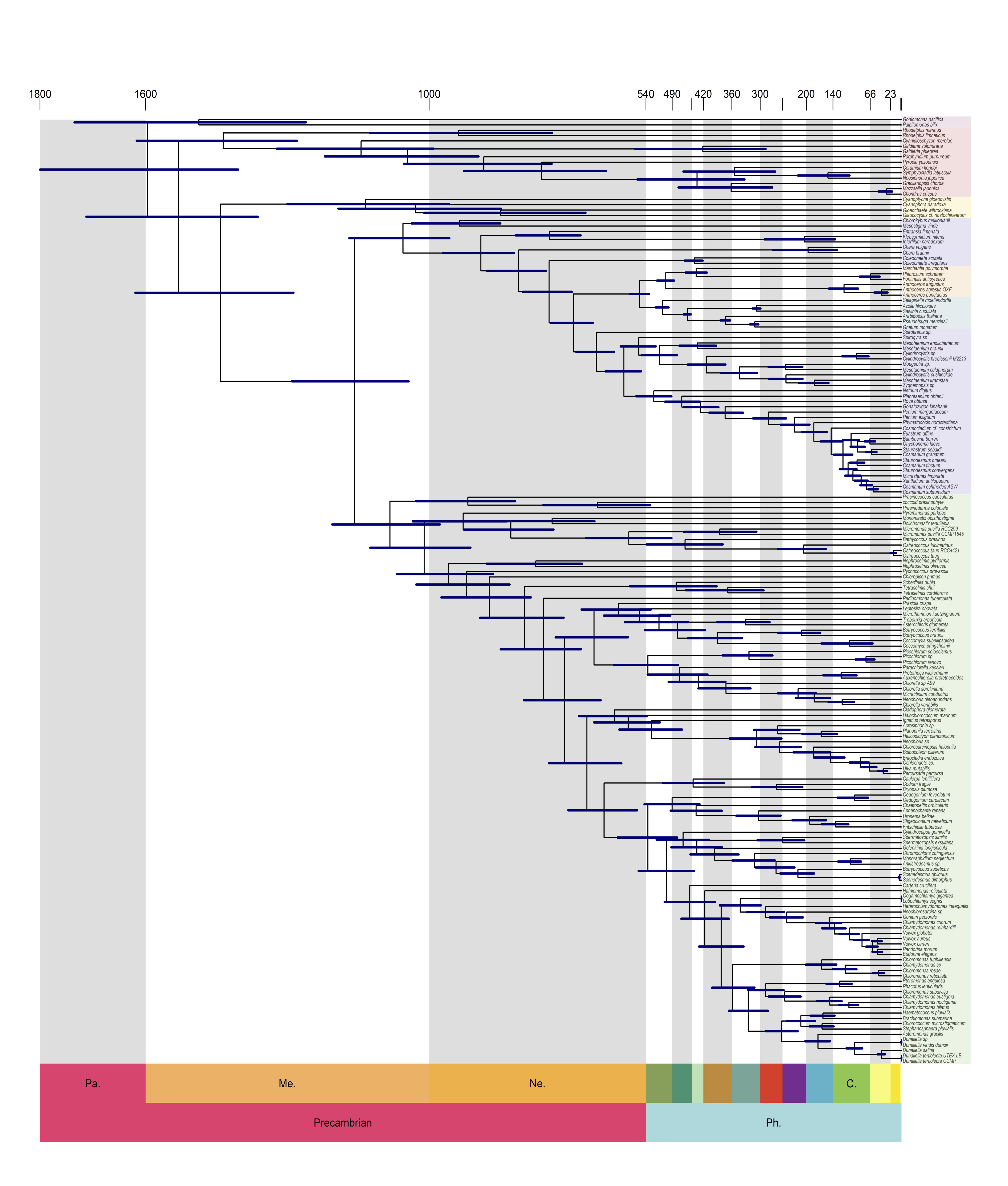
**

**Supplementary Information 6:** Time calibrated phylogeny of Archaeplastida with 95% HPD age uncertainties, using correlated rates. Branches and taxa colour coded by lineage: Outgroup (Dusty red), Rhodophytes (Red), Glaucophytes (Yellow), Chlorophytes (Green), Streptophytes (Purple), Bryophytes (Blue), Tracheophytes (Orange).

**
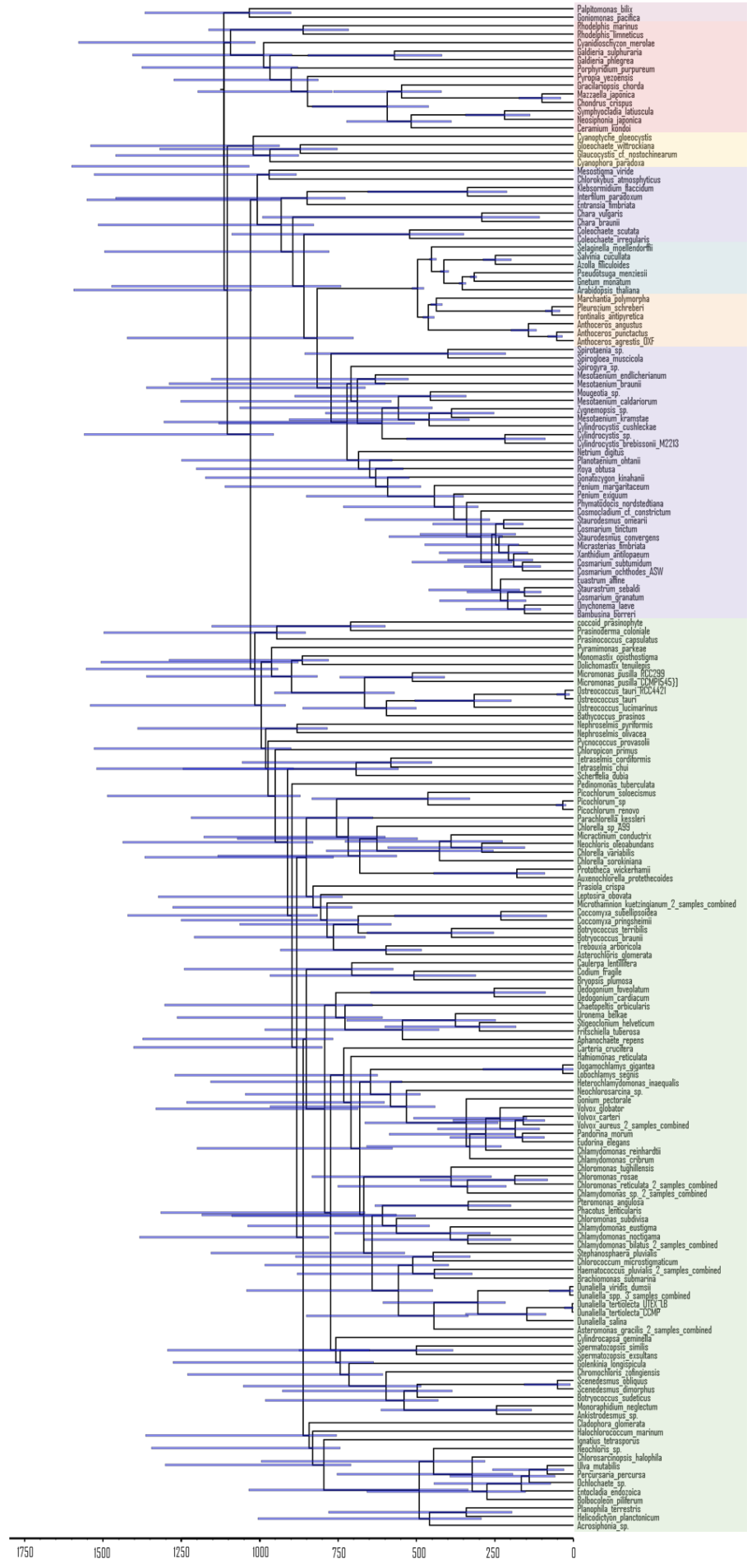
**

**Supplementary Information 7:** Time calibrated phylogeny of Archaeplastida with 95% HPD age uncertainties, using Phylobayes. Branches and taxa colour coded by lineage: Outgroup (Dusty red), Rhodophytes (Red), Glaucophytes (Yellow), Chlorophytes (Green), Streptophytes (Purple), Bryophytes (Blue), Tracheophytes (Orange).

**
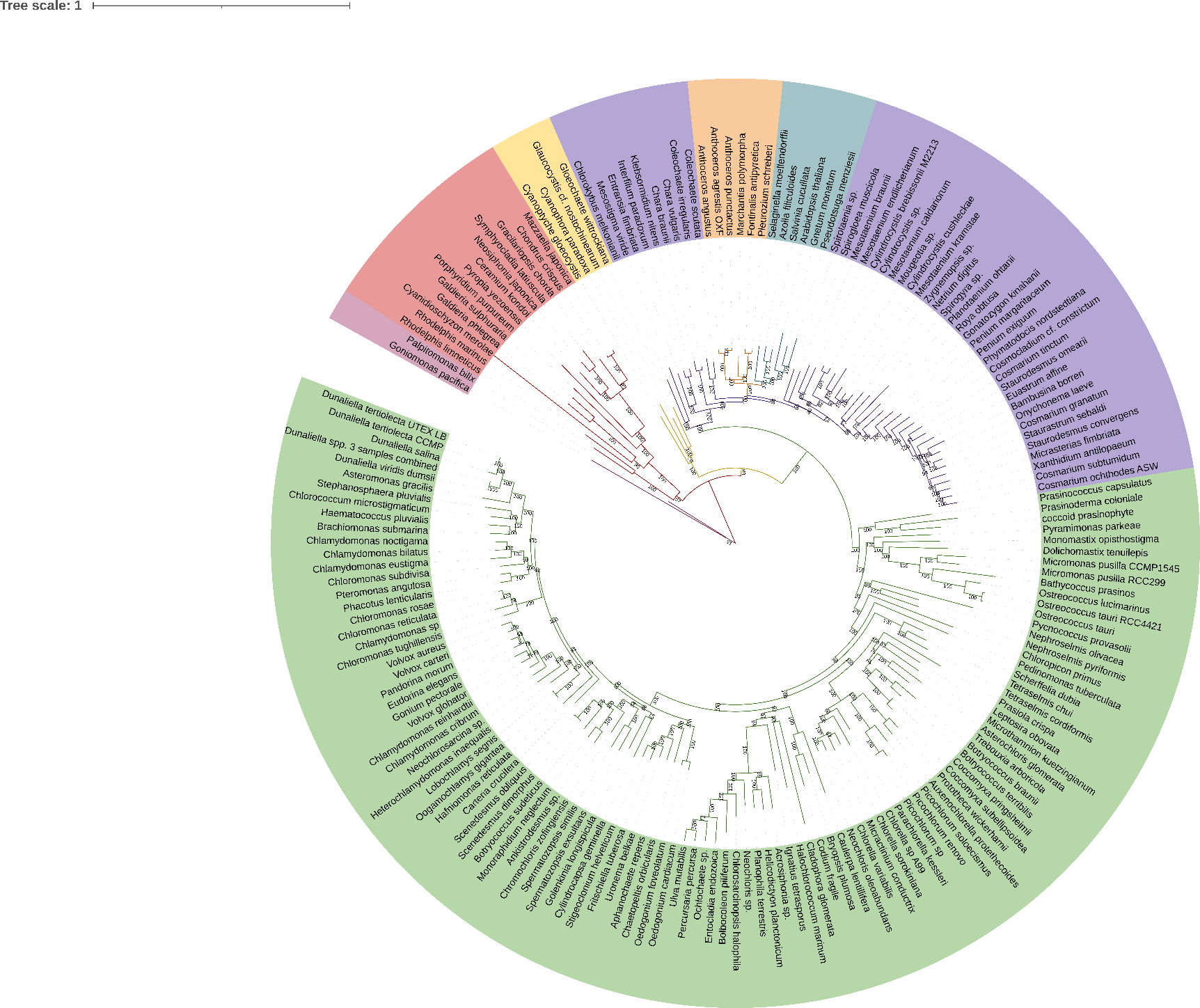
**

**Supplementary Information 8:** Phylogeny of Archaeplastida based on a 50% reduced amino acid dataset to test compositional heterogeneity. Branches and taxa colour coded by lineage: Outgroup (Dusty red), Rhodophytes (Red), Glaucophytes (Yellow), Chlorophytes (Green), Streptophytes (Purple), Bryophytes (Blue), Tracheophytes (Orange).

***

***

**Supplementary Information 9:** Nodes in archaeplastid phylogeny referred to in Supplementary Information 10.

**Supplementary Information 10:** The posterior divergence time estimates (95% HPD) for all nodes with reference to the tree in SI 9, supplied in units of 100 million years. Key nodes have been provided with a taxonomic classification.

| Classification | Node | Posterior mean | (95% HPD-CI) |
| --- | --- | --- | --- |
|  | t_n179 | 15.6238 | (14.0882-17.5095) |
|  | t_n180 | 13.9833 | (11.8666-16.4027) |
| Archaeplastida | t_n181 | 15.3278 | (13.8746-17.1246) |
| Rhodophyta | t_n182 | 14.2877 | (12.8448-16.1298) |
|  | t_n183 | 7.0203 | (5.2933-9.1655) |
|  | t_n184 | 11.5561 | (10.0812-13.1042) |
|  | t_n185 | 10.569 | (9.2298-12.0485) |
|  | t_n186 | 3.649 | (2.7213-4.8150) |
|  | t_n187 | 8.9901 | (7.7168-10.3467) |
| Bangiophyceae/Florideophyceae | t_n188 | 7.7485 | (6.5533-9.0180) |
| Florideophyceae | t_n189 | 4.6186 | (3.8594-5.3925) |
|  | t_n190 | 3.75 | (3.0523-4.5130) |
|  | t_n191 | 0.2844 | (0.2042-0.3852) |
|  | t_n192 | 3.7706 | (3.0990-4.5080) |
|  | t_n193 | 1.8434 | (1.4164-2.3733) |
| Viridiplantae & Glaucophyta | t_n194 | 14.5325 | (13.1730-16.2480) |
| Glaucophyta | t_n195 | 6.7264 | (5.7547-8.2080) |
|  | t_n196 | 5.9768 | (5.0497-7.3897) |
|  | t_n197 | 4.7724 | (3.8530-6.1659) |
| Viridiplantae | t_n198 | 12.5736 | (11.4782-13.6945) |
| Streptophyta | t_n199 | 10.936 | (9.9892-11.9929) |
|  | t_n200 | 9.6981 | (8.7303-10.8061) |
| Phragmoplastophyta & Klebsormidiophyceae | t_n201 | 8.9627 | (8.2125-9.7661) |
|  | t_n202 | 7.3227 | (6.3138-8.2534) |
|  | t_n203 | 1.1516 | (0.8081-1.5468) |
| Phragmoplastophyta | t_n204 | 8.2921 | (7.5996-9.0132) |
|  | t_n205 | 0.8857 | (0.6363-1.1719) |
|  | t_n206 | 7.7571 | (7.1044-8.4293) |
|  | t_n207 | 4.339 | (4.1988-4.5754) |
| Anydrophyta | t_n208 | 7.3336 | (6.7098-7.9666) |
| Embryophyta | t_n209 | 5.5656 | (5.2672-5.8650) |
| Tracheophyta | t_n210 | 5.0557 | (4.8397-5.2558) |
| Euphyllophyta | t_n211 | 4.4795 | (4.3523-4.5560) |
|  | t_n212 | 3.1158 | (3.0315-3.2584) |
| Spermatophyta | t_n213 | 3.7397 | (3.5719-3.9126) |
|  | t_n214 | 3.1225 | (3.0382-3.2687) |
| Bryophytes | t_n215 | 4.8432 | (4.4341-5.2412) |
|  | t_n216 | 0.3903 | (0.2991-0.4955) |
|  | t_n217 | 0.1296 | (0.0914-0.1768) |
|  | t_n218 | 4.1474 | (3.7095-4.6186) |
|  | t_n219 | 0.4355 | (0.3239-0.5701) |
| Zygnematophyceae | t_n220 | 6.9563 | (6.3336-7.5632) |
|  | t_n221 | 6.506 | (5.9001-7.0763) |
|  | t_n222 | 6.112 | (5.5065-6.6778) |
| Zygnematales | t_n223 | 5.5869 | (4.9970-6.1411) |
|  | t_n224 | 4.7385 | (4.1465-5.3447) |
|  | t_n225 | 4.2035 | (3.6494-4.7544) |
|  | t_n226 | 0.8773 | (0.6553-1.1221) |
|  | t_n227 | 3.3874 | (2.9564-3.9031) |
|  | t_n228 | 2.4491 | (2.0466-2.9941) |
|  | t_n229 | 2.0593 | (1.6485-2.4637) |
|  | t_n230 | 1.5283 | (1.1699-1.9111) |
| Desmidiales | t_n231 | 6.0611 | (5.4689-6.6181) |
|  | t_n232 | 5.616 | (5.0416-6.1538) |
|  | t_n233 | 5.2843 | (4.7109-5.8025) |
|  | t_n234 | 4.8534 | (4.2974-5.3400) |
|  | t_n235 | 4.2216 | (3.6715-4.6642) |
|  | t_n236 | 3.7528 | (3.2161-4.1638) |
|  | t_n237 | 3.3346 | (2.8380-3.7003) |
|  | t_n238 | 2.9267 | (2.4677-3.2615) |
|  | t_n239 | 2.5727 | (2.1735-2.8886) |
|  | t_n240 | 2.2534 | (1.8918-2.5528) |
|  | t_n241 | 1.9547 | (1.6368-2.2347) |
|  | t_n242 | 1.2897 | (1.0153-1.5751) |
|  | t_n243 | 1.4012 | (1.1291-1.6639) |
|  | t_n244 | 2.3582 | (1.9826-2.6611) |
|  | t_n245 | 1.9189 | (1.5831-2.2309) |
|  | t_n246 | 2.1502 | (1.8044-2.4399) |
|  | t_n247 | 1.7947 | (1.5024-2.0665) |
|  | t_n248 | 1.5602 | (1.2851-1.8259) |
|  | t_n249 | 1.2311 | (0.9819-1.4865) |
| Chlorophytes | t_n250 | 11.9394 | (10.8801-12.9711) |
|  | t_n251 | 9.2996 | (7.9354-10.5812) |
|  | t_n252 | 6.0638 | (4.6870-7.6470) |
|  | t_n253 | 11.3165 | (10.2589-12.3627) |
| Mamiellophyceae | t_n254 | 10.1785 | (9.2336-11.2083) |
|  | t_n255 | 8.9255 | (7.9943-9.9840) |
|  | t_n256 | 7.8617 | (6.9087-8.9204) |
|  | t_n257 | 6.14 | (5.3396-7.0715) |
|  | t_n258 | 3.6435 | (2.8786-4.4749) |
|  | t_n259 | 4.7994 | (4.0535-5.7031) |
|  | t_n260 | 2.0081 | (1.5223-2.5217) |
|  | t_n261 | 0.1781 | (0.1284-0.2377) |
|  | t_n262 | 10.8444 | (9.7790-11.8658) |
|  | t_n263 | 7.8316 | (6.5202-9.1211) |
|  | t_n264 | 10.4809 | (9.4061-11.4912) |
|  | t_n265 | 10.0172 | (8.9073-10.9973) |
| Core chlorophytes | t_n266 | 9.3756 | (7.8087-10.3330) |
|  | t_n267 | 3.9694 | (3.0812-4.8010) |
|  | t_n268 | 3.0336 | (2.2359-3.8152) |
|  | t_n269 | 8.9786 | (7.2884-9.9041) |
| Trebouxio-/Ulvo-/Chloro-phyceae | t_n270 | 8.5558 | (6.7540-9.4423) |
| Trebouxiophyceae | t_n271 | 7.6098 | (6.0656-8.3805) |
|  | t_n272 | 6.8685 | (5.5161-7.5811) |
|  | t_n273 | 6.2693 | (4.9924-6.9513) |
|  | t_n274 | 5.7707 | (4.5136-6.3983) |
|  | t_n275 | 5.3046 | (4.1461-5.9100) |
|  | t_n276 | 3.617 | (2.8218-4.2587) |
|  | t_n277 | 4.3349 | (3.4000-4.8878) |
|  | t_n278 | 1.0622 | (0.5890-1.7275) |
|  | t_n279 | 2.4931 | (1.9041-2.8692) |
|  | t_n280 | 5.97 | (4.9220-6.6598) |
|  | t_n281 | 2.8787 | (2.1875-3.5350) |
|  | t_n282 | 0.2892 | (0.2096-0.3737) |
|  | t_n283 | 5.0381 | (4.2177-5.6457) |
|  | t_n284 | 4.5576 | (3.8410-5.1385) |
|  | t_n285 | 1.6229 | (1.2580-2.0378) |
|  | t_n286 | 3.8568 | (3.2377-4.4535) |
|  | t_n287 | 2.2064 | (1.8220-2.5953) |
|  | t_n288 | 1.8568 | (1.5057-2.2095) |
|  | t_n289 | 1.2816 | (0.9940-1.6095) |
| Ulvophyceae/Chlorophyceae | t_n290 | 8.1182 | (6.3557-8.9578) |
| Ulvophyceae | t_n291 | 7.4697 | (5.7884-8.2914) |
|  | t_n292 | 7.1041 | (5.4893-7.9112) |
|  | t_n293 | 6.5448 | (5.0472-7.2951) |
|  | t_n294 | 5.0341 | (3.8406-5.6459) |
|  | t_n295 | 4.1907 | (3.1397-4.7819) |
|  | t_n296 | 2.5601 | (1.9106-3.1927) |
|  | t_n297 | 4.4714 | (3.3642-5.0573) |
|  | t_n298 | 3.7049 | (2.8138-4.2091) |
| Ulvales | t_n299 | 3.166 | (2.3752-3.6449) |
|  | t_n300 | 2.2628 | (1.7241-2.6042) |
|  | t_n301 | 1.8908 | (1.4236-2.2100) |
|  | t_n302 | 1.1027 | (0.7963-1.3503) |
| Chlorophyceae | t_n303 | 7.6841 | (5.9362-8.4681) |
| Bryopsidales | t_n304 | 5.2607 | (4.1309-6.0941) |
|  | t_n305 | 2.9851 | (2.2006-3.6910) |
|  | t_n306 | 6.8153 | (5.0039-7.5034) |
| Chaetophorales/Oedogoniales | t_n307 | 5.9288 | (4.4133-6.6190) |
|  | t_n308 | 0.9142 | (0.6729-1.2119) |
|  | t_n309 | 5.2184 | (3.8889-5.8682) |
|  | t_n310 | 3.4704 | (2.8005-4.1016) |
|  | t_n311 | 2.1416 | (1.7504-2.5453) |
|  | t_n312 | 1.5586 | (1.2475-1.9004) |
|  | t_n313 | 6.3921 | (4.6478-7.0581) |
|  | t_n314 | 5.8854 | (4.2504-6.5136) |
|  | t_n315 | 5.5331 | (3.9621-6.1259) |
|  | t_n316 | 5.1455 | (3.6614-5.7212) |
|  | t_n317 | 4.5878 | (3.2391-5.1549) |
|  | t_n318 | 0.0012 | (0.0002-0.0033) |
|  | t_n319 | 3.9152 | (2.7918-4.4543) |
|  | t_n320 | 3.365 | (2.3876-3.8531) |
|  | t_n321 | 2.3837 | (1.8246-2.7432) |
|  | t_n322 | 2.2447 | (1.7219-2.5862) |
|  | t_n323 | 1.857 | (1.4248-2.1501) |
|  | t_n324 | 1.4823 | (1.1249-1.7289) |
|  | t_n325 | 1.1513 | (0.8739-1.3566) |
|  | t_n326 | 0.9202 | (0.6840-1.1172) |
|  | t_n327 | 0.945 | (0.7046-1.1423) |
|  | t_n328 | 4.8421 | (3.4305-5.3840) |
|  | t_n329 | 2.2044 | (1.7228-2.6151) |
|  | t_n330 | 1.5703 | (1.2140-1.9268) |
|  | t_n331 | 0.6066 | (0.4419-0.7746) |
|  | t_n332 | 4.4391 | (3.1342-4.9449) |
|  | t_n333 | 3.882 | (2.7526-4.3845) |
|  | t_n334 | 1.6023 | (1.1340-2.0365) |
|  | t_n335 | 3.3211 | (2.3104-3.7898) |
|  | t_n336 | 2.108 | (1.4571-2.4707) |
|  | t_n337 | 1.4985 | (1.0101-1.8400) |
|  | t_n338 | 3.6483 | (2.6313-4.0965) |
|  | t_n339 | 3.0271 | (2.1459-3.4180) |
|  | t_n340 | 2.2929 | (1.5591-2.6613) |
|  | t_n341 | 2.2703 | (1.6072-2.6733) |
|  | t_n342 | 2.5091 | (1.7898-2.9198) |
|  | t_n343 | 1.3336 | (0.9706-1.6171) |
|  | t_n344 | 0.0071 | (0.0000-0.0311) |
|  | t_n345 | 0.4824 | (0.3570-0.6213) |
|  | t_n346 | 0.0005 | (0.0000-0.0017) |
|  | t_n347 | 5.9088 | (4.1891-6.5248) |
|  | t_n348 | 5.5261 | (3.8416-6.1160) |
|  | t_n349 | 3.083 | (2.2267-3.7303) |
|  | t_n350 | 5.0262 | (3.4415-5.6002) |
|  | t_n351 | 4.0527 | (2.7325-4.5758) |
|  | t_n352 | 3.4795 | (2.2971-3.9905) |
|  | t_n353 | 2.8143 | (1.7689-3.3032) |
|  | t_n354 | 0.0617 | (0.0436-0.0841) |
|  | t_n355 | 1.5159 | (1.0114-1.9287) |

***References***

1. O’Leary, N.A., Wright, M.W., Brister, J.R., Ciufo, S., Haddad, D., McVeigh, R., Rajput, B., Robbertse, B., Smith-White, B., Ako-Adjei, D., *et al.* (2016). Reference sequence (RefSeq) database at NCBI: current status, taxonomic expansion, and functional annotation. Nucleic Acids Res. *44*, 733–745.

2. Gawryluk, R.M.R., Tikhonenkov, D. V., Hehenberger, E., Husnik, F., Mylnikov, A.P., and Keeling, P.J. (2019). Non-photosynthetic predators are sister to red algae. Nature *572*, 240–243.

3. Schönknecht, G., Chen, W.H., Ternes, C.M., Barbier, G.G., Shrestha, R.P., Stanke, M., Bräutigam, A., Baker, B.J., Banfield, J.F., Garavito, R.M., *et al.* (2013). Gene transfer from bacteria and archaea facilitated evolution of an extremophilic eukaryote. Science (80-. ). *339*, 1207–1210.

4. Li, L., Wang, S., Wang, H., Sahu, S.K., Marin, B., Li, H., Xu, Y., Liang, H., Li, Z., Cheng, S., *et al.* (2020). The genome of Prasinoderma coloniale unveils the existence of a third phylum within green plants. Nat. Ecol. Evol. *4*, 1220–1231.

5. Qiu, H., Price, D.C., Weber, A.P.M., Reeb, V., Chan Yang, E., Lee, J.M., Kim, S.Y., Yoon, H.S., and Bhattacharya, D. (2013). Adaptation through horizontal gene transfer in the cryptoendolithic red alga Galdieria phlegrea. Curr. Biol. *23*, 865–866.

6. Leebens-Mack, J.H., Barker, M.S., Carpenter, E.J., Deyholos, M.K., Gitzendanner, M.A., Graham, S.W., Grosse, I., Li, Z., Melkonian, M., Mirarab, S., *et al.* (2019). One thousand plant transcriptomes and the phylogenomics of green plants. Nature *574*, 679–685.

7. Bhattacharya, D., Price, D.C., Xin Chan, C., Qiu, H., Rose, N., Ball, S., Weber, A.P.M.M., Cecilia Arias, M., Henrissat, B., Coutinho, P.M., *et al.* (2013). Genome of the red alga Porphyridium purpureum. Nat. Commun. *4*.

8. Collén, J., Porcel, B., Carré, W., Ball, S.G., Chaparro, C., Tonon, T., Barbeyron, T., Michel, G., Noel, B., Valentin, K., *et al.* (2013). Genome structure and metabolic features in the red seaweed Chondrus crispus shed light on evolution of the Archaeplastida. Proc. Natl. Acad. Sci. U. S. A. *110*, 5247–5252.

9. Price, D.C., Chan, C.X., Yoon, H.S., Yang, E.C., Qiu, H., Weber, A.P.M., Schwacke, R., Gross, J., Blouin, N.A., Lane, C., *et al.* (2012). Cyanophora paradoxa genome elucidates origin of photosynthesis in algae and plants. Science (80-. ). *335*, 843–847.

10. Moreau, H., Verhelst, B., Couloux, A., Derelle, E., Rombauts, S., Grimsley, N., Van Bel, M., Poulain, J., Katinka, M., Hohmann-Marriott, M.F., *et al.* (2012). Gene functionalities and genome structure in Bathycoccus prasinos reflect cellular specializations at the base of the green lineage. Genome Biol. *13*.

11. Palenik, B., Grimwood, J., Aerts, A., Rouzé, P., Salamov, A., Putnam, N., Dupont, C., Jorgensen, R., Derelle, E., Rombauts, S., *et al.* (2007). The tiny eukaryote Ostreococcus provides genomic insights into the paradox of plankton speciation. Proc. Natl. Acad. Sci. U. S. A. *104*, 7705–7710.

12. Derelle, E., Ferraz, C., Rombauts, S., Rouzé, P., Worden, A.Z., Robbens, S., Partensky, F., Degroeve, S., Echeynié, S., Cooke, R., *et al.* (2006). Genome analysis of the smallest free-living eukaryote Ostreococcus tauri unveils many unique features. Proc. Natl. Acad. Sci. U. S. A. *103*, 11647–11652.

13. Worden, A.Z., Lee, J.H., Mock, T., Rouzé, P., Simmons, M.P., Aerts, A.L., Allen, A.E., Cuvelier, M.L., Derelle, E., Everett, M. V., *et al.* (2009). Green evolution and dynamic adaptations revealed by genomes of the marine picoeukaryotes micromonas. Science (80-. ). *324*, 268–272.

14. Lemieux, C., Turmel, M., Otis, C., and Pombert, J.-F. (2019). A streamlined and predominantly diploid genome in the tiny marine green alga Chloropicon primus. Nat. Commun. *10*.

15. Armaleo, D., Müller, O., Lutzoni, F., Andrésson, Ó.S., Blanc, G., Bode, H.B., Collart, F.R., Grande, F.D., Dietrich, F., Grigoriev, I. V., *et al.* (2019). The lichen symbiosis re-viewed through the genomes of Cladonia grayi and its algal partner Asterochloris glomerata. BMC Genomics *20*.

16. Gao, C., Wang, Y., Shen, Y., Yan, D., He, X., Dai, J., and Wu, Q. (2014). Oil accumulation mechanisms of the oleaginous microalga Chlorella protothecoides revealed through its genome, transcriptomes, and proteomes. BMC Genomics *15*, 582.

17. Browne, D.R., Jenkins, J., Schmutz, J., Shu, S., Barry, K., Grimwood, J., Chiniquy, J., Sharma, A., Niehaus, T.D., Weiss, T.L., *et al.* (2017). Draft nuclear genome sequence of the liquid hydrocarbon-accumulating green microalga botryococcus braunii race B (Showa). Genome Announc. *5*.

18. Blanc, G., Duncan, G., Agarkova, I., Borodovsky, M., Gurnon, J., Kuo, A., Lindquist, E., Lucas, S., Pangilinan, J., Polle, J., *et al.* (2010). The Chlorella variabilis NC64A genome reveals adaptation to photosymbiosis, coevolution with viruses, and cryptic sex. Plant Cell *22*, 2943–2955.

19. Blanc, G., Agarkova, I., Grimwood, J., Kuo, A., Brueggeman, A., Dunigan, D.D., Gurnon, J., Ladunga, I., Lindquist, E., Lucas, S., *et al.* (2012). The genome of the polar eukaryotic microalga Coccomyxa subellipsoidea reveals traits of cold adaptation. Genome Biol. *13*.

20. Pombert, J.F., Blouin, N.A., Lane, C., Boucias, D., and Keeling, P.J. (2014). A Lack of Parasitic Reduction in the Obligate Parasitic Green Alga Helicosporidium. PLoS Genet. *10*.

21. Arriola, M.B., Velmurugan, N., Zhang, Y., Plunkett, M.H., Hondzo, H., and Barney, B.M. (2017). Genome sequences of Chlorella sorokiniana UTEX 1602 and Micractinium conductrix SAG 241.80: implications to maltose excretion by a green alga. Plant J. *93*, 566–586.

22. Foflonker, F., Price, D.C., Qiu, H., Palenik, B., Wang, S., and Bhattacharya, D. (2015). Genome of the halotolerant green alga Picochlorum sp. reveals strategies for thriving under fluctuating environmental conditions. Environ. Microbiol. *17*, 412–426.

23. Gonzalez-Esquer, C.R., Twary, S.N., Hovde, B.T., and Starkenburg, S.R. (2018). Nuclear, Chloroplast, and Mitochondrial Genome Sequences of the Prospective Microalgal Biofuel Strain Picochlorum soloecismus. Genome Announc. *6*, 1498–15017.

24. Hirooka, S., Hirose, Y., Kanesaki, Y., Higuchi, S., Fujiwara, T., Onuma, R., Era, A., Ohbayashi, R., Uzuka, A., Nozaki, H., *et al.* (2017). Acidophilic green algal genome provides insights into adaptation to an acidic environment. Proc. Natl. Acad. Sci. U. S. A. *114*, 8304–8313.

25. Azua-Bustos, A., González-Silva, C., Arenas-Fajardo, C., and Vicuña, R. (2012). Extreme environments as potential drivers of convergent evolution by exaptation: The Atacama Desert Coastal Range case. Front. Microbiol. *3*.

26. Hanschen, E.R., Marriage, T.N., Ferris, P.J., Hamaji, T., Toyoda, A., Fujiyama, A., Neme, R., Noguchi, H., Minakuchi, Y., Suzuki, M., *et al.* (2016). The Gonium pectorale genome demonstrates co-option of cell cycle regulation during the evolution of multicellularity. Nat. Commun. *7*, 11370.

27. Featherston, J., Arakaki, Y., Hanschen, E.R., Ferris, P.J., Michod, R.E., Olson, B.J.S.C., Nozaki, H., and Durand, P.M. (2018). The 4-celled tetrabaena socialis nuclear genome reveals the essential components for genetic control of cell number at the origin of multicellularity in the volvocine lineage. Mol. Biol. Evol. *35*, 855–870.

28. Prochnik, S.E., Umen, J., Nedelcu, A.M., Hallmann, A., Miller, S.M., Nishii, I., Ferris, P., Kuo, A., Mitros, T., Fritz-Laylin, L.K., *et al.* (2010). Genomic analysis of organismal complexity in the multicellular green alga volvox carteri. Science (80-. ). *329*, 223–226.

29. Wang, S., Li, L., Li, H., Sahu, S.K., Wang, H., Xu, Y., Xian, W., Song, B., Liang, H., Cheng, S., *et al.* (2019). Genomes of early-diverging streptophyte algae shed light on plant terrestrialization. Nat. Plants *6*, 95–106.

30. Roth, M.S., Cokus, S.J., Gallaher, S.D., Walter, A., Lopez, D., Erickson, E., Endelman, B., Westcott, D., Larabell, C.A., Merchant, S.S., *et al.* (2017). Chromosome-level genome assembly and transcriptome of the green alga Chromochloris zofingiensis illuminates astaxanthin production. Proc. Natl. Acad. Sci. U. S. A. *114*, 4296–4305.

31. Hori, K., Maruyama, F., Fujisawa, T., Togashi, T., Yamamoto, N., Seo, M., Sato, S., Yamada, T., Mori, H., Tajima, N., *et al.* (2014). Klebsormidium flaccidum genome reveals primary factors for plant terrestrial adaptation. Nat. Commun. *5*, 3978.

32. Bogen, C., Al-Dilaimi, A., Albersmeier, A., Wichmann, J., Grundmann, M., Rupp, O., Lauersen, K.J., Blifernez-Klassen, O., Kalinowski, J., Goesmann, A., *et al.* (2013). Reconstruction of the lipid metabolism for the microalga Monoraphidium neglectum from its genome sequence reveals characteristics suitable for biofuel production. BMC Genomics *14*.

33. Dasgupta, C.N., Nayaka, S., Toppo, K., Singh, A.K., Deshpande, U., and Mohapatra, A. (2018). Draft genome sequence and detailed characterization of biofuel production by oleaginous microalga Scenedesmus quadricauda LWG002611. Biotechnol. biofuels *11*.

34. Cheng, S., Xian, W., Fu, Y., Marin, B., Keller, J., Wu, T., Sun, W., Li, X., Xu, Y., Zhang, Y., *et al.* (2019). Genomes of Subaerial Zygnematophyceae Provide Insights into Land Plant Evolution. Cell *179*, 1057–1067.

35. Arimoto, A., Nishitsuji, K., Higa, Y., Arakaki, N., Hisata, K., Shinzato, C., Satoh, N., and Shoguchi, E. (2019). A siphonous macroalgal genome suggests convergent functions of homeobox genes in algae and land plants. DNA Res. *26*, 183–192.

36. Matsuzaki, M., Misumi, O., Shin-I, T., Maruyama, S., Takahara, M., Miyagishima, S.Y., Mori, T., Nishida, K., Yagisawa, F., Nishida, K., *et al.* (2004). Genome sequence of the ultrasmall unicellular red alga Cyanidioschyzon merolae 10D. Nature *428*, 653–657.

37. De Clerck, O., Kao, S.-M., Bogaert, K.A., Blomme, J., Foflonker, F., Kwantes, M., Vancaester, E., Vanderstraeten, L., Aydogdu, E., Boesger, J., *et al.* (2018). Insights into the Evolution of Multicellularity from the Sea Lettuce Genome. Curr. Biol. *28*, 2921–2933.

38. Jiao, C., Sørensen, I., Sun, X., Sun, H., Behar, H., Alseekh, S., Philippe, G., Palacio Lopez, K., Sun, L., Reed, R., *et al.* (2020). The Penium margaritaceum Genome: Hallmarks of the Origins of Land Plants. Cell *181*, 1097–1111.

39. Nishiyama, T., Sakayama, H., de Vries, J., Buschmann, H., Saint-Marcoux, D., Ullrich, K.K., Haas, F.B., Vanderstraeten, L., Becker, D., Lang, D., *et al.* (2018). The Chara Genome: Secondary Complexity and Implications for Plant Terrestrialization. Cell *174*, 448–464.

40. Yu, J., Li, L., Wang, S., Dong, S., Chen, Z., Patel, N., Goffinet, B., Chen, H., Liu, H., and Liu, Y. (2020). Draft genome of the aquatic moss Fontinalis antipyretica (Fontinalaceae, Bryophyta). Gigabyte *2020*, 1–9.

41. Zhang, J., Fu, X.X., Li, R.Q., Zhao, X., Liu, Y., Li, M.H., Zwaenepoel, A., Ma, H., Goffinet, B., Guan, Y.L., *et al.* (2020). The hornwort genome and early land plant evolution. Nat. Plants *6*, 107–118.

42. Bowman, J.L., Kohchi, T., Yamato, K.T., Jenkins, J., Shu, S., Ishizaki, K., Yamaoka, S., Nishihama, R., Nakamura, Y., Berger, F., *et al.* (2017). Insights into Land Plant Evolution Garnered from the Marchantia polymorpha Genome. Cell *171*, 287–304.

43. Li, F., Nishiyama, T., Waller, M., Frangedakis, E., Keller, J., Li, Z., Fernandez-Pozo, N., Barker, M.S., Bennett, T., Blázquez, M.A., *et al.* (2020). Anthoceros genomes illuminate the origin of land plants and the unique biology of hornworts. Nat. Plants *6*, 259–272.

44. Rensing, S.A., Lang, D., Zimmer, A.D., Terry, A., Salamov, A., Shapiro, H., Nishiyama, T., Perroud, P.-F., Lindquist, E.A., Kamisugi, Y., *et al.* (2008). The Physcomitrella Genome Reveals Evolutionary Insights into the Conquest of Land by Plants. Science (80-. ). *319*, 64–69.

45. Li, F.-W., Brouwer, P., Carretero-Paulet, L., Cheng, S., de Vries, J., Delaux, P.-M., Eily, A., Koppers, N., Kuo, L.-Y., Li, Z., *et al.* (2018). Fern genomes elucidate land plant evolution and cyanobacterial symbioses. Nat. Plants *4*, 460–472.

46. Pederson, E.R.A., Warshan, D., and Rasmussen, U. (2019). Genome sequencing of Pleurozium schreberi: The assembled and annotated draft genome of a pleurocarpous feather moss. G3 Genes, Genomes, Genet. *9*, 2791–2797.

47. Wan, T., Liu, Z., Leitch, I.J., Xin, H., Maggs-Kölling, G., Gong, Y., Li, Z., Marais, E., Liao, Y., Dai, C., *et al.* (2021). The Welwitschia genome reveals a unique biology underpinning extreme longevity in deserts. Nat. Commun. *12*.

48. Banks, J.A.J.A., Nishiyama, T., Hasebe, M., Bowman, J.L., Gribskov, M., dePamphilis, C., Albert, V.A., Aono, N., Aoyama, T., Ambrose, B.A., *et al.* (2011). The Selaginella Genome Identifies Genetic Changes Associated with the Evolution of Vascular Plants. Science (80-. ). *332*, 960–963.

49. Neale, D.B., McGuire, P.E., Wheeler, N.C., Stevens, K.A., Crepeau, M.W., Cardeno, C., Zimin, A. V., Puiu, D., Pertea, G.M., Sezen, U.U., *et al.* (2017). The Douglas-Fir genome sequence reveals specialization of the photosynthetic apparatus in Pinaceae. G3 Genes, Genomes, Genet. *7*, 3157–3167.

50. Brawley, S.H., Blouin, N.A., Ficko-Blean, E., Wheeler, G.L., Lohr, M., Goodson, H. V., Jenkins, J.W., Blaby-Haas, C.E., Helliwell, K.E., Chan, C.X., *et al.* (2017). Insights into the red algae and eukaryotic evolution from the genome of Porphyra umbilicalis (Bangiophyceae, Rhodophyta). Proc. Natl. Acad. Sci. U. S. A. *114*, 6361–6370.

51. Kaul, S., Koo, H.L., Jenkins, J., Rizzo, M., Rooney, T., Tallon, L.J., Feldblyum, T., Nierman, W., Benito, M.I., Lin, X., *et al.* (2000). Analysis of the genome sequence of the flowering plant Arabidopsis thaliana. Nature *408*, 796–815.

52. Lee, J.M., Yang, E.C., Graf, L., Yang, J.H., Qiu, H., Zelzion, U., Chan, C.X., Stephens, T.G., Weber, A.P.M., Boo, G.H., *et al.* (2018). Analysis of the draft genome of the red seaweed gracilariopsis chorda provides insights into genome size evolution in rhodophyta. Mol. Biol. Evol. *35*, 1869–1886.

53. Gibson, T.M., Shih, P.M., Cumming, V.M., Fischer, W.W., Crockford, P.W., Hodgskiss, M.S.W., Wörndle, S., Creaser, R.A., Rainbird, R.H., Skulski, T.M., *et al.* (2018). Precise age of Bangiomorpha pubescens dates the origin of eukaryotic photosynthesis. Geology *46*, 135–138.

54. Fralick, P., Davis, D., and S, K. (2002). The age of the Gunflint Formation, Ontario, Canada: Single zircon U-Pb age determinations from reworked volcanic ash. Can. J. Earth Sci. *39*, 1085–1091.

55. Butterfield, N.J. (2000). Bangiomorpha pubescens n. gen., n. sp.: implications for the evolution of sex, multicellularity, and the Mesoproterozoic/Neoproterozoic radiation of eukaryotes. Paleobiology *26*, 386–404.

56. Xiao, S., Knoll, A.H., Yuan, X., and Pueschel, C.M. (2004). Phosphatized multicellular algae in the Neoproterozoic Doushantuo Formation, China, and the early evolution of florideophyte red algae. Am. J. Bot. *91*, 214–27.

57. YANG, C., ROONEY, A.D., CONDON, D.J., LI, X.-H., GRAZHDANKIN, D. V., BOWYER, F.T., HU, C., MACDONALD, F.A., and ZHU, M. (2021). The tempo of Ediacaran evolution. Sci. Adv. *7*.

58. Tang, Q., Pang, K., Yuan, X., and Xiao, S. (2020). A one-billion-year-old multicellular chlorophyte. Nat. Ecol. Evol. *4*, 543–549.

59. Harris, B.J., Clark, J.W., Schrempf, D., Szöllősi, G.J., Donoghue, P.C.J., Hetherington, A.M., and Williams, T.A. (2022). Divergent evolutionary trajectories of bryophytes and tracheophytes from a complex common ancestor of land plants. Nat. Ecol. Evol.

60. Mastik, V., and Tinn, O. (2015). New dasycladalean algal species from the Kalana Lagerstätte (Silurian, Estonia). J. Paleontol. *89*.

61. Morris, J.L., Puttick, M.N., Clark, J.W., Edwards, D., Kenrick, P., Pressel, S., Wellman, C.H., Yang, Z., Schneider, H., and Donoghue, P.C.J. (2018). The timescale of early land plant evolution. Proc. Natl. Acad. Sci. *115*, 2274–2283.

62. Colbath, G.K., and Grenfell, H.R. (1995). Review of biological affinities of Paleozoic acid-resistant, organic-walled eukaryotic algal microfossils (including “acritarchs”). Rev. Palaeobot. Palynol. *86*.

63. Volkman, J.K. (2014). Acyclic isoprenoid biomarkers and evolution of biosynthetic pathways in green microalgae of the genus Botryococcus. Org. Geochem. *75*.

64. Adam, P., Schaeffer, P., and Albrecht, P. (2006). C40 monoaromatic lycopane derivatives as indicators of the contribution of the alga Botryococcus braunii race L to the organic matter of Messel oil shale (Eocene, Germany). Org. Geochem. *37*.

65. Traverse, A. (1955). Occurrence of the Oil-Forming Alga Botryococcus in Lignites and Other Tertiary Sediments. Micropaleontology *1*.

66. Gradstein, F.M., Ogg, J.G., Smith, A.G., Bleeker, W., and Lourens, L.J. (2004). A new Geologic Time Scale, with special reference to Precambrian and Neogene. Episodes *27*.

67. Tennant, R.K., Lux, T.M., Sambles, C.M., Kuhn, N.J., Petticrew, E.L., Oldfield, R., Parker, D.A., Hatton, J., Moore, K.A., Lee, R., *et al.* (2019). Palaeogenomics of the Hydrocarbon Producing Microalga Botryococcus braunii. Sci. Rep. *9*.

68. Rubinstein, C. V., Gerrienne, P., de la Puente, G.S., Astini, R.A., and Steemans, P. (2010). Early Middle Ordovician evidence for land plants in Argentina (eastern Gondwana). New Phytol. *188*, 365–369.

69. Cenci, U., Sibbald, S.J., Curtis, B.A., Kamikawa, R., Eme, L., Moog, D., Henrissat, B., Maréchal, E., Chabi, M., Djemiel, C., *et al.* (2018). Nuclear genome sequence of the plastid-lacking cryptomonad Goniomonas avonlea provides insights into the evolution of secondary plastids. BMC Biol. *16*.

70. Hassoun, M., Wynne, M.J., Moussa, H., Salhi, G., Zbakh, H., Riadi, H., and Kazzaz, M. (2018). An investigation of members of the tribe Ceramieae (Ceramiaceae, Rhodophyta) occurring on both the Mediterranean and Atlantic shores of Morocco. Algae *33*, 243–267.

71. Kim, M.S., and Yang, E.C. (2006). Taxonomy and Phylogeny of Neosiphonia japonica (Rhodomelaceae, Rhodophyta) Based on rbcL and cpeA/B Gene Sequences. Algae *21*, 287–294.

72. Xu, X., Yanga, H., Khalil, Z.G., Yin, L., Xiao, X., Salim, A.A., Song, F., and Capon, R.J. (2019). Bromocatechol conjugates from a Chinese marine red alga, Symphyocladia latiuscula. Pytochemistry *158*, 20–25.

73. Choi, H.-G. (2001). Morphology and Reproduction of Heterosiphonia pulchra and H. japonica (Ceramiales, Rhodophyta). Algae *16*, 387–409.

74. Collén, J., Porcel, B., Carré, W., Ball, S.G., Chaparro, C., Tonon, T., Barbeyron, T., Michel, G., Noel, B., Valentin, K., *et al.* (2013). Genome structure and metabolic features in the red seaweed Chondrus crispus shed light on evolution of the Archaeplastida. Proc. Natl. Acad. Sci. U. S. A. *110*, 5247–5252.

75. Tian, Y., Liu, Y., Qi, X., Zhang, X., and Wang, H. (2018). Early development and life history of Mazzaella japonica (Mikami) Hommersand. J. Appl. Phycol. *30*, 1933–1941.

76. Tang, Y., Liu, H., Yu, Y., and Li, X. (2016). Early life stage development of Gloiopeltis furcata (Gigartinales, Endocladiaceae) from northern China. J. Biotechnol. Res. *7*, 49–56.

77. Zhao, F., Zhao, Z., and Liu, J. (2014). Effects of temperature on the early development of carpospores of Ahnfeltiopsis flabelliformis (Gigartinales, Rhodophyta). Arch. Hydrobiol. Suppl. Algol. Stud. *145*–*146*, 181–193.

78. Kang, P.J., An, J.W., and Nam, K.W. (2018). New record of Dumontia contorta and D. alaskana (Dumontiaceae, Gigartinales) in Korea. Fish. Aquat. Sci. *21*.

79. Hurtado, A.Q., and Cheney, D.P. (2005). Propagule Production of Eucheuma denticulatum (Burman) Collins et Harvey by Tissue Culture. Bot. Mar. *46*, 338–341.

80. Rudke, A.R., Andrade, C.J. de, and Ferreira, S.R.S. (2020). Kappaphycus alvarezii macroalgae: An unexplored and valuable biomass for green biorefinery conversion. Trends Food Sci. Technol. *103*, 214–224.

81. Dumilag, R. V., Liao, L.M., and Lluisma, A.O. (2014). Phylogeny of Betaphycus (Gigartinales, Rhodophyta) as inferred from COI sequences and morphological observations on B. philippinensis. J. Appl. Phycol. *26*, 587–595.

82. Wong, P.-F., Tan, L.-J., Nawi, H., and AbuBakar, S. (2006). PROTEOMICS OF THE RED ALGA, GRACILARIA CHANGII (GRACILARIALES, RHODOPHYTA). J. Phycol. *42*, 113–120.

83. Wang, H.W., Kawaguchi, S., Horiguchi, T., and Masuda, M. (2019). Reinstatement of Grateloupia catenata (Rhodophyta, Halymeniaceae) on the basis of morphology and rbcL sequences. Phycologia *39*.

84. Mikami, K., Li, C., Irie, R., and Hama, Y. (2019). A unique life cycle transition in the red seaweed Pyropia yezoensis depends on apospory. Commun. Biol. *2*.

85. Desquilbet, T.E., Duval, J.-C., Robert, B., Houmard, J., and Thomas, J.C. (2003). In the Unicellular Red Alga Rhodella violacea Iron Deficiency Induces an Accumulation of Uncoupled LHC. Plant Cell Physiol. *44*, 1141–1151.

86. Wilson, S.M., Pickett-Heaps, J.D., and West, J.A. (2006). Vesicle transport and the cytoskeleton in the unicellular red alga Glaucosphaera vacuolata. Phycol. Res. *54*, 15–20.

87. Zuccarello, G., West, J., Bitans, A., and Kraft, G. (2019). Molecular phylogeny of Rhodochaete parvula (Bangiophycidae, Rhodophyta). Phycologia *39*, 75–81.

88. Daglio, Y., Romero, J.M., Lagorio, M.G., Stortz, C.A., and Rodríguez, M.C. (2021). Mixed approach on Chroodactylon ornatum (Stylonematophyceae, Rhodophyta) tolerance to hyposalinity: growth, photosynthetic performance and carbohydrate analysis. Phycologia *61*, 16–26.

89. Rossoni, A.W., Price, D.C., Seger, M., Lyska, D., Lammers, P., Bhattacharya, D., and Weber, A.P. (2019). The genomes of polyextremophilic cyanidiales contain 1% horizontally transferred genes with diverse adaptive functions. Elife *8*.

90. Price, D.C., Goodenough, U.W., Roth, R., Lee, J.-H., Kariyawasam, T., Mutwil, M., Ferrari, C., Facchinelli, F., Ball, S.G., Cenci, U., *et al.* (2019). Analysis of an improved Cyanophora paradoxa genome assembly. DNA Res. *26*, 287–299.

91. Miyashita, H., Ikemoto, H., Kurano, N., Miyachi, S., and Chihara, M. (1993). PRASINOCOCCUS CAPSULATUS GEN. ET SP. NOV., A NEW MARINE COCCOID PRASINOPHYTE. J. Gen. Appl. Microbiol. *39*, 571–582.

92. Li, L., Wang, S., Wang, H., Sahu, S.K., Marin, B., Li, H., Xu, Y., Liang, H., Li, Z., Cheng, S., *et al.* (2020). The genome of Prasinoderma coloniale unveils the existence of a third phylum within green plants. Nat. Ecol. Evol. *4*, 1220–1231.

93. Yau, S., Santos, A.L. dos, Eikrem, W., Ribeiro, C.G., Gourvil, P., Balzano, S., Escande, M.-L., Moreau, H., and Vaulot, D. (2019). Mantoniella beaufortii and Mantoniella baffinensis sp. nov. (Mamiellales, Mamiellophyceae), two new green algal species from the high arctic. J. Phycol. *56*, 37–51.

94. Nelson, D.R., Khraiwesh, B., Fu, W., Alseekh, S., Jaiswal, A., Chaiboonchoe, A., Hazzouri, K.M., O’Connor, M.J., Butterfoss, G.L., Drou, N., *et al.* (2017). The genome and phenome of the green alga chloroidium sp. UTEX 3007 reveal adaptive traits for desert acclimatization. Elife *6*.

95. Repetti, S.I., Iha, C., Uthanumallian, K., Jackson, C.J., Chen, Y., Chan, C.X., and Verbruggen, H. (2021). Nuclear genome of a pedinophyte pinpoints genomic innovation and streamlining in the green algae. New Phytol. *233*, 2144–2154.

96. Hou, Z., Ma, X., Shi, X., Li, X., Yang, L., Xiao, S., De Clerck, O., Leliaert, F., and Zhong, B. (2022). Phylotranscriptomic insights into a Mesoproterozoic–Neoproterozoic origin and early radiation of green seaweeds (Ulvophyceae). Nat. Commun. *13*.

97. Li, X., Hou, Z., Xu, C., Shi, X., Yang, L., Lewis, L.A., and Zhong, B. (2021). Large Phylogenomic Data sets Reveal Deep Relationships and Trait Evolution in Chlorophyte Green Algae. Genome Biol. Evol. *13*.

98. Herron, M.D., Rashidi, A., Shelton, D.E., and Driscoll, W.W. (2013). Cellular differentiation and individuality in the ‘minor’ multicellular taxa. Biol. Rev. *88*, 844–861.

99. Richter, D., Matuła, J., Urbaniak, J., Waleron, M., and Czerwik-Marcinkowska, J. (2017). Molecular, morphological and ultrastructural characteristics of Prasiola crispa (Lightfoot) Kützing (Chlorophyta) from Spitsbergen (Arctic). Polar Biol. *40*, 379–397.

100. Sivakumar, G., Jeong, K., and Jackson O Lay, J. (2014). Bioprocessing of Stichococcus bacillaris strain siva2011. Biotechnol. biofuels *7*.

101. Bakuła, Z., Siedlecki, P., Gromadka, R., Gawor, J., Gromadka, A., Pomorski, J.J., Panagiotopoulou, H., and Jagielski, T. (2021). A first insight into the genome of Prototheca wickerhamii, a major causative agent of human protothecosis. BMC Genomics *22*.

102. Tartar, A. (2013). The Non-Photosynthetic Algae Helicosporidium spp.: Emergence of a Novel Group of Insect Pathogens. Insects *4*, 375–391.

103. Foflonker, F., Mollegard, D., Ong, M., Yoon, H.S., and Bhattacharya, D. (2018). Genomic Analysis of Picochlorum Species Reveals How Microalgae May Adapt to Variable Environments. Mol. Biol. Evol. *35*, 2702–2711.

104. S, K., P, H., and Malik, A. (2022). Chlorella minutissima as a functional food: evaluation on nutritional profile and antioxidant potential of the metabolites. Biomass Convers. Biorefinery.

105. Safi, C., Pollio, A., and Olivieri, G. (2021). Neochloris oleoabundans from nature to industry: a comprehensive review. Rev. Environ. Sci. Bio/Technology *20*, 943–958.

106. Li, X., Hou, Z., Xu, C., Shi, X., Yang, L., Lewis, L.A., and Zhong, B. (2021). Large phylogenomic datasets reveal deep relationships and trait evolution in chlorophyte green algae. Genome Biol. Evol. *13*.

107. Zhu, H., Hu, Y., Liu, F., Hu, Z., and Liu, G. (2019). Characterization of the Chloroplast Genome of Trentepohlia odorata (Trentepohliales, Chlorophyta), and Discussion of its Taxonomy. Int. J. Mol. Sci. *20*.

108. Goldberg, W.M., Makemson, J.C., and Colley, S.B. (1984). Entocladia endozoica sp. nov., A Pathogenic Chlorophyte: Structure, Life History, Physiology, and Effect on Its Coral Host. Biol. Bull. *166*, 368–383.

109. Wichard, T., Charrier, B., Mineur, F., Bothwell, J.H., Clerck, O. De, and Coates, J.C. (2015). The green seaweed Ulva: a model system to study morphogenesis. Front. Plant Sci. *6*.

110. O’Kelly, C.J., Bellows, W.K., and Wysor, B. (2004). PHYLOGENETIC POSITION OF BOLBOCOLEON PILIFERUM (ULVOPHYCEAE, CHLOROPHYTA): EVIDENCE FROM REPRODUCTION, ZOOSPORE AND GAMETE ULTRASTRUCTURE, AND SMALL SUBUNIT RRNA GENE SEQUENCES. J. Phycol. *40*, 209–222.

111. Hall, J.D., Sheath, R.G., McCourt, R.M., and Stancheva, R. (2017). Ochlochaete incrustans sp. nov., a new species of freshwater ulvophycean algae from California, USA, with notes on Friedaea torrenticola. Phycologia *57*, 465–476.

112. Pratt, S., Lundquist, C., Nelson, W., and Gemmill, C. (2012). A new record of Percursaria percursa (Ulvaceae, Ulvales) on the North Island, New Zealand. New Zeal. J. Bot. *51*, 71–74.

113. Watanabe, S., Maiwa, N., and Kuroda, F. (2001). Phylogenetic status of Helicodictyon planctonicum and Desmochloris halophila gen. et comb. nov. and the definition of the class Ulvophyceae (Chlorophyta). Phycologia *40*, 421–434.

114. Deason, T.R., Silva, P.C., Watanabe, S., and Floyd, G.L. (1991). Taxonomic status of the species of the green algal genus Neochloris. Plant Syst. Evol. *177*, 213–219.

115. Wujek, D.E., and Thompson, R.H. (1999). The Algal Genera Chaetopeltis, Oligochaetophora, and Polychaetophora (Chaetopeltidales, Chlorophyta). Trans. Kansas Acad. Sci. *102*, 40–46.

116. Su, H., Feng, J., LV, J.-P., Liu, Q., Nan, F.-R., and Xie, S.-L. (2019). Fritschiella aquatilis (Chaetophoraceae, Chlorophyta), a new freshwater green algae species from China. Phytotaxa *392*.

117. Skinner, S., and Entwisle, T.J. (2004). Non-marine algae of Australia: 5. Macroscopic Chaetophoraceae (Chaetophorales, Chlorophyta). Telopea *10*.

118. Leliaert, F., Rueness, J., Boedeker, C., Maggs, C.A., Cocquyt, E., Verbruggen, H., and Clerck, O. De (2009). Systematics of the marine microfilamentous green algae Uronema curvatum and Urospora microscopica (Chlorophyta). J. Phycol. *44*, 487–496.

119. Ducoff, H.S., Butler, B.D., and Geffon, E.J. (1964). X-Ray Survival Studies on the Alga Brachiomonas submarina Bohlin. Radiat. Res. *23*, 446–453.

120. Archibald, P.A. (1988). Chlorococcum pamirum and C. salinum, two new species of the Chlorophyceae from Central Asia. Br. Phycol. J. *23*, 121–128.

121. H, M., T, N., K, N., H, N., and M, T. (2015). Phylogenetic Position and Molecular Chronology of a Colonial Green Flagellate, Stephanosphaera pluvialis (Volvocales, Chlorophyceae), among Unicellular Algae. J. Eukaryot. Microbiol. *63*, 340–348.

122. Matsuzaki, R., Suzuki, S., Yamaguchi, H., Kawachi, M., Kanesaki, Y., Yoshikawa, H., Mori, T., and Nozaki, H. (2021). The Rubisco small subunits in the green algal genus Chloromonas provide insights into evolutionary loss of the eukaryotic carbon-concentrating organelle, the pyrenoid. BMC Ecol. Evol. *21*.

123. Schlegel, I., Krienitz, L., and Hepperle, D. (2000). Variability of calcification of Phacotus lenticularis (Chlorophyta, Chlamydomonadales) in nature and culture. Phycologia *39*, 318–322.

124. Herron, M.D., and Michod, R.E. (2007). EVOLUTION OF COMPLEXITY IN THE VOLVOCINE ALGAE: TRANSITIONS IN INDIVIDUALITY THROUGH DARWIN’S EYE. Evolution (N. Y). *62*.

125. Helliwell, K.E., Pandhal, J., Cooper, M.B., Longworth, J., Kudahl, U.J., Russo, D.A., Tomsett, E. V., Bunbury, F., Salmon, D.L., Smirnoff, N., *et al.* (2017). Quantitative proteomics of a B12-dependent alga grown in coculture with bacteria reveals metabolic tradeoffs required for mutualism. New Phytol. *217*, 599–612.

126. Watanabe, S., Mitsui, K., Nakayama, T., and Inouye, I. (2006). PHYLOGENETIC RELATIONSHIPS AND TAXONOMY OF SARCINOID GREEN ALGAE: CHLOROSARCINOPSIS, DESMOTETRA, SARCINOCHLAMYS GEN. NOV., NEOCHLOROSARCINA, AND CHLOROSPHAEROPSIS (CHLOROPHYCEAE, CHLOROPHYTA). J. Phycol. *42*, 679–695.

127. Nakazawa, A., Krienitz, L., and Nozaki, H. (2010). Taxonomy of the unicellular green algal genus Vitreochlamys (Volvocales), based on comparative morphology of cultured material. Eur. J. Phycol. *36*.

128. Susanti, H., and Nakayama, T. (2021). Study of a green algae Lobochlamys segnis Strain-019 from peatland. IOP Conf. Ser. Earth Environ. Sci. *948*.

129. Silva, J. da, and Drysdale, V.L. (2018). Isogamy in large and complex volvocine algae is consistent with the gamete competition theory of the evolution of anisogamy. Proc. R. Soc. B *285*.

130. Nakada, T., Suda, S., and Nozaki, H. (2007). A TAXONOMIC STUDY OF HAFNIOMONAS (CHLOROPHYCEAE) BASED ON A COMPARATIVE EXAMINATION OF CULTURED MATERIAL. J. Phycol. *43*, 397–411.

131. Watanabe, S. (2020). Reclassification of Chlamydomonas monticola as Heterochlamydomonas (Volvocales, Chlorophyceae). Phycol. Res. *68*, 332–335.

132. Sluiman, H.J. (1985). MITOSIS AND CELL DIVISION IN CYLINDROCAPSA GEMINELLA (CHLOROPHYCEAE). J. Phycol. *21*, 523–532.

133. Hazen, T.E. (1902). The Ulothricaceae and Chaetophoraceae of the United States. In Memoirs of the Torrey Botanical Club, pp. 135–245.

134. Zhang, Y., Fe, Y., Bai, F., and Liu, J. (2021). The oleaginous astaxanthin-producing alga Chromochloris zofingiensis: potential from production to an emerging model for studying lipid metabolism and carotenogenesis. Biotechnoloy for Biofuels *14*.

135. Senousy, H.H., Beakes, G.W., and Hack, E. (2004). PHYLOGENETIC PLACEMENT OF BOTRYOCOCCUS BRAUNII (TREBOUXIOPHYCEAE) ANDBOTRYOCOCCUS SUDETICUS ISOLATE UTEX 2629 (CHLOROPHYCEAE). J. Phycol. *40*, 412–423.

136. Schomaker, R.A., and Dudycha, J.L. (2021). De novo transcriptome assembly of the green alga Ankistrodesmus falcatus. PLoS One *16*.

137. Vannini, C., Domingo, G., Marsoni, M., Mattia, F. De, Labra, M., Castiglioni, S., and Bracale, M. (2011). Effects of a complex mixture of therapeutic drugs on unicellular algae Pseudokirchneriella subcapitata. Aquat. Toxicol. *101*, 459–465.

138. El-Sheekh, M.M., El-Naggar, A.H., Osman, M.E.H., and El-Mazaly, E. (2003). Effect of cobalt on growth, pigments and the photosynthetic electron transport in Monoraphidium minutum and Nitzchia perminuta. Brazilian J. Plant Physiol. *15*, 159–166.

139. Gontcharov, A.A., and Melkonian, M. (2003). Unusual position of the genus Spirotaenia (Zygnematophyceae) among streptophytes revealed by SSU rDNA and rbcL sequence comparisons. Phycologia *43*, 105–113.

140. Herburger, K., Karsten, U., and Holzinger, A. (2016). Entransia and Hormidiella, sister lineages of Klebsormidium (Streptophyta), respond differently to light, temperature, and desiccation stress. Protoplasma *253*, 1309–1323.

141. Bansod, V.I., and Patil, N.H. (2019). DIVERSITY OF GENUS COSMARIUM FROM BODALKASA DAM, GONDIA DISTRICT, MAHARASHTRA, INDIA. J. Emerg. Technol. Innov. Res. *6*.

142. Mikhailyuk, T., Holzinger, A., Massalski, A., and Karsten, U. (2014). Morphology and ultrastructure of Interfilum and Klebsormidium (Klebsormidiales, Streptophyta) with special reference to cell division and thallus formation. Eur. J. Phycol. *49*, 395–412.

143. Turmel, M., Otis, C., and Lemieux, C. (2005). The complete chloroplast DNA sequences of the charophycean green algae Staurastrum and Zygnema reveal that the chloroplast genome underwent extensive changes during the evolution of the Zygnematales. BMC Biol. *3*.

144. Turmel, M., Otis, C., and Lemieux, C. (2006). The Chloroplast Genome Sequence of Chara vulgaris Sheds New Light into the Closest Green Algal Relatives of Land Plants. Mol. Biol. Evol. *23*, 1324–1338.

145. Hall, J.D., McCourt, R.M., and Delwiche, C.F. (2008). Patterns of cell division in the filamentous Desmidiaceae, close green algal relatives of land plants. Am. J. Bot. *95*, 643–654.

146. Brook, A.J. (1981). Calcium sulphate inclusions in the desmids Bambusina and Gonatozygon. Eur. J. Phycol. *16*, 267–272.

147. Anissimova, O. V., and Staer, O. V. (2018). Morphology of Cell Wall Pore Channels in the Genus Euastrum Ralfs (Desmidiales). Moscow Univ. Biol. Sci. Bull. *73*, 28–31.

148. Stamenkovic, M., Woelken, E., and Hanelt, D. (2014). Ultrastructure of Cosmarium strains (Zygnematophyceae, Streptophyta) collected from various geographic locations shows species-specific differences both at optimal and stress temperatures. Protoplasma *251*, 1491–1509.

149. Domozych, D.S., Elliott, L., Kiemle, S.N., and Gretz, M.R. (2007). Pleurotaenium trabecula, a desmid of wetland biofilms: the extracellular matrix and adhesion mechanisms. J. Phycol. *43*, 1022–1038.

150. Neustupa, J., Št’astný, J., and Škaloud, P. (2014). Splitting of Micrasterias fimbriata (Desmidiales, Viridiplantae) into two monophyletic species and description of Micrasterias compereana sp. nov. Plant Ecol. Evol. *147*, 405–411.

151. Taniguchi, G.M., Peres, A.C., Senna, P.A.C., and Compère, P. (2003). The Desmid Genera Cosmarium, Actinotaenium and Cosmocladium from an Oxbow Lake, Jataí Ecological Station (Southeastern Brazil). Syst. Geogr. Plants *73*, 133–159.

152. Frohne, W.C. (1942). Notes on Phymatodocis nordstedtiana Wolle (Desmidiaceae) from South Carolina and Its var. Minor Børgesen from Georgia. Trans. Am. Microsc. Soc. *61*, 438–441.

153. Domozych, D.S. (2014). Penium margaritaceum: A Unicellular Model Organism for Studying Plant Cell Wall Architecture and Dynamics. Plants (Basel) *3*, 543–558.

154. Hirano, N., Marukawa, Y., Abe, J., Hashiba, S., Ichikawa, M., Tanabe, Y., Ito, M., Nishii, I., Tsuchikane, Y., and Sekimoto, H. (2015). A Receptor-Like Kinase, Related to Cell Wall Sensor of Higher Plants, is Required for Sexual Reproduction in the Unicellular Charophycean Alga, Closterium peracerosum-strigosum-littorale Complex. Plant Cell Physiol. *56*, 1456–62.

155. Cortona, A. Del, Jackson, C.J., Bucchini, F., Bel, M. Van, D’hondt, S., Škaloud, P., Delwiche, C.F., Knoll, A.H., Raven, J.A., Verbruggen, H., *et al.* (2020). Neoproterozoic origin and multiple transitions to macroscopic growth in green seaweeds. Proc. Natl. Acad. Sci. *117*, 2551–2559.

156. Gontcharov, A.A., and Melkonian, M. (2010). MOLECULAR PHYLOGENY AND REVISION OF THE GENUS NETRIUM (ZYGNEMATOPHYCEAE, STREPTOPHYTA): NUCLEOTAENIUM GEN. NOV. J. Phycol. *46*, 346–362.

157. Eder, M., and Lütz-Meindl, U. (2010). Analyses and localization of pectin-like carbohydrates in cell wall and mucilage of the green alga Netrium digitus. Protoplasma *243*, 25–38.

158. MORRIS, G.J., COULSON, G.E., and ENGELS, M. (1986). A Cryomicroscopic Study of Cylindrocystis brebissonii De Bary and Two Species of Micrasterias Ralfs (Conjugatophyceae, Chiorophyta) during Freezing and Thawing. J. Exp. Bot. *37*, 842–856.

159. Morand, L.Z., Kidd, D.G., and Lagarias, J.C. (1993). Phytochrome Levels in the Green Alga Mesotaenium caldariorum Are Light Regulated. Plant Physiol. *101*, 97–103.

160. Pichrtová, M., Holzinger, A., Kulichová, J., Ryšánek, D., Šoljaková, T., Trumhová, K., and Nemcova, Y. (2018). Molecular and morphological diversity of Zygnema and Zygnemopsis (Zygnematophyceae, Streptophyta) from Svalbard (High Arctic). Eur. J. Phycol. *53*, 492–508.

161. Brickley, M.R., Weise, V., Hawes, C., and Cobb, A.H. (2010). Morphology and dynamics of mitochondria in Mougeotia sp. Eur. J. Phycol. *45*, 258–266.

162. Vogel, V., and Bergmann, P. (2018). Culture of Spirogyra sp. in a flat-panel airlift photobioreactor. 3 Biotech *8*.

163. Pires, N., and Dolan, L. (2012). Morphological evolution in land plants: new designs with old genes. Philos. Trans. R. Soc. B Biol. Sci. *367*, 508–518.
